# Mechanics of the Spatiotemporal Evolution of Sulcal Pits in the Folding Brain

**DOI:** 10.1002/hbm.70332

**Published:** 2025-08-27

**Authors:** Akbar Solhtalab, Yanchen Guo, Ali Gholipour, Weiying Dai, Mir Jalil Razavi

**Affiliations:** ^1^ Department of Mechanical Engineering State University of New York at Binghamton Binghamton New York USA; ^2^ Department of Computer Science State University of New York at Binghamton Binghamton New York USA; ^3^ Department of Radiological Sciences University of California Irvine Irvine California USA; ^4^ Department of Electrical Engineering and Computer Science University of California Irvine Irvine California USA; ^5^ Department of Radiology Boston Children's Hospital Boston Massachusetts USA

**Keywords:** brain folding, fetal brain, finite element simulation, mechanics, sulcal pits

## Abstract

Understanding the development of complex brain surface morphologies during the fetal stage is essential for uncovering mechanisms underlying brain disorders linked to abnormal cortical folding. However, knowledge of the spatiotemporal evolution of fetal brain landmarks remains limited due to the lack of longitudinal data capturing multiple timepoints for individual brains. In this study, we develop and validate an image‐based true‐scale mechanical model to investigate the spatiotemporal evolution of brain sulcal pits in individual fetal brains. Altered sulcal pits patterns have been observed in disorders such as autism spectrum disorder (ASD), polymicrogyria, Down syndrome, and agenesis of the corpus callosum. Our model, constructed using magnetic resonance imaging (MRI) scans from the first timepoint of longitudinal data, predicts the brain's surface morphology by comparing the distribution of sulcal pits between the predicted models and MRI scans from a later timepoint. This dynamic model simulates how a smooth fetal brain with primary folds evolves into a convoluted morphology. Our results align with imaging data, showing that sulcal pits are stable during brain development and can serve as key markers linking prenatal and postnatal brain characteristics. The model provides a platform for future hypothesis testing and for studying the effects of mechanical parameters on the evolution of sulcal pits in both healthy and disordered brains. This research represents a significant advancement in understanding fetal brain development and its connection to disorders that manifest as abnormal sulcal pit patterns later in life.

## Graphical abstract

This study introduces a true‐scale, image‐based mechanical model to investigate the spatiotemporal evolution of sulcal pits during fetal brain development. The model predicts cortical surface morphology, demonstrating the stability of sulcal pits over time and providing key insights into brain folding mechanisms and their relevance to neurodevelopmental disorders.

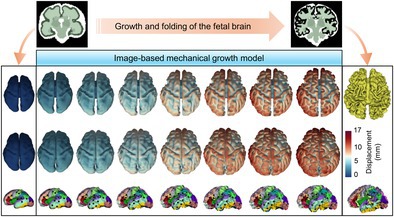

## 1 Introduction

Cortical folding, also known as gyrification, is a complex process through which the brain develops convolutions on the surface of the cerebral cortex (Zilles et al. 2013; Striedter et al. 2015; Fernández et al. 2016; Welker 1990). This folding results in the formation of gyri (ridges) and sulci (valleys), significantly increasing the surface area of the cortex and enhancing cognitive functions as well as efficient information processing (Ten Donkelaar 2023). Cortical folding plays a vital role in the cognitive abilities of the human brain (Del‐Valle‐Anton and Borrell 2022; Sun and Hevner 2014). Abnormalities in this process have been linked to various neurodevelopmental and psychiatric disorders, which underscores its importance in understanding brain development and function (Hardan et al. 2004; Harris et al. 2004; Choi et al. 2022). Typically, cortical folding begins during the third trimester of gestation and continues to develop after birth. Initially, primary folds form between 20 and 25 gestational weeks, followed by the emergence of secondary folds between 34 and 36 weeks, and tertiary folds that develop after 36 weeks on the surface of the brain (Fernández and Borrell 2023). Primary folds, which are primarily influenced by genetic factors, tend to be more stable than secondary and tertiary folds, which exhibit greater variability across individual brains (Ronan and Fletcher 2015).

The variability in folding morphologies among individual brains makes it challenging to study the underlying mechanisms of cortical folding. However, recent brain imaging studies (Im and Grant 2019) have highlighted that “*sulcal pits*” (Régis et al. 2005; Lohmann et al. 2008; Im et al. 2010) have the potential to serve as valuable indicators for studying cortical maturation and detecting altered cortical development during the early stages of neurodevelopment. Sulcal pits, which are the first deep sulcal folds, are under strong spatiotemporal genetic control and their spatial distribution exhibits a relatively stable pattern from infancy (Meng et al. 2014). Emerging first in utero and reaching their greatest depth in the adult brain, sulcal pits are remarkable anatomical features closely associated with the cytoarchitectonic protomap and human brain function (Welker 1990; Lohmann et al. 2008; Dubois et al. 2008; Yun et al. 2020; Zilles et al. 1997; Hasnain 2001; Eickhoff et al. 2006; Fischl et al. 2008). Evidence indicates that, at term birth, the consistent spatial distributions of sulcal pits in major sulci across individuals are already established and remain relatively stable during the first 2 years of life (Meng et al. 2014; Auzias et al. 2015). Notably, these sulcal pits are genetically controlled during early fetal development (Fukuchi‐Shimogori and Grove 2001; Miyashita‐Lin et al. 1999; Rakic 1988, 2001), their depths have a heritable basis (Le Guen et al. 2018), and sulcal graphs exhibit greater similarity in twin pairs compared to unrelated individuals (Im et al. 2011). Therefore, sulcal pits provide a crucial avenue for exploring disruptions in topological patterning, connectivity, and brain function simultaneously, while mitigating challenges associated with individual variability in folding patterns. In postnatal brains with cerebral malformations, sulcal patterns differ significantly from those in normal brains, both in location and depth (Im et al. 2017). The timing of sulcal pit appearance is positively correlated with heritability estimates, with recent findings suggesting that these estimates for sulcal pits decrease linearly over the evolutionary timeline of gyrification (Huang et al. 2023). This suggests that cortical folds formed earlier in the gyrification process are influenced more strongly by genetic factors than those formed later. However, much like the surface morphology of cortical folds, the spatiotemporal evolution of sulcal pits and their stability within individual brains remain poorly understood due to a lack of sufficient longitudinal imaging data of individual brains (Im and Grant 2019).

Numerous studies have demonstrated the interplay between genetically guided biological processes and physical forces in orchestrating cortical growth and folding (Bayly et al. 2014; Budday et al. 2014; Razavi, Zhang, Li, et al. 2015; Hilgetag and Barbas 2006; de Vareilles et al. 2023). As emerging cortical layers develop, variations in cellular density and differential growth rates create mechanical forces that drive the convolution and folding of the cerebral cortex (Garcia et al. 2018). The differential tangential growth (DTG) theory posits that the faster growth of outer brain layers compared to the slower growth of inner layers initiates mechanical instability, which ultimately leads to cortical folding (Caviness 1975). This leading hypothesis is supported by several experimental and computational studies (Tallinen et al. 2014, 2016; Razavi, Zhang, Liu, and Wang 2015; Bayly et al. 2013; Balouchzadeh et al. 2023; Budday, Raybaud, and Kuhl 2015; Zhang et al. 2017; Wang et al. 2022, 2021; Darayi et al. 2022). In addition to the DTG theory, other factors such as radial constraint (Rakic 1995), cranial constraint (Chen et al. 2010), and axonal tension (Essen 1997; Van Essen 2020; Garcia et al. 2021; Chavoshnejad et al. 2021, 2023; Holland et al. 2015) have also been proposed to play roles in the formation and modulation of cortical folds during brain development.

To date, many biomechanical models developed to study brain folding have primarily relied on simplified and idealized shapes or focused solely on adjusting individual parameters to investigate the growth and folding of the human brain (Bayly et al. 2013; Balouchzadeh et al. 2023; Darayi et al. 2022; Toro and Burnod 2005; Budday, Steinmann, et al. 2015; Campos et al. 2020; Leyva‐Mendivil et al. 2015; Wang et al. 2019, 2020; Jafarabadi et al. 2023). Moreover, there has been a lack of opportunities to compare the surface morphologies produced by these models with the intricate complexities observed in actual individual brain anatomies. While these models contribute to our basic understanding of brain folding mechanics, they fall short of providing a comprehensive representation of the true‐scale nature of brain growth and folding. Therefore, a robust and comprehensive model is needed to effectively capture the complex evolution of brain surface morphology and sulcal pits.

In this study, we develop image‐based true‐scale models to simulate brain growth and folding, providing insights into the emergence and evolution of cortical folds and sulcal pits. We refer to a true‐scale model as a subject‐specific computational model constructed directly from MRI data, preserving the anatomical size and geometry of the fetal brain. We synergistically extend our analysis by integrating magnetic resonance imaging (MRI) data. Recent advancements in super‐resolution reconstruction techniques applied to T_2_‐weighted fetal brain structural MRI (Gholipour et al. 2010; Kuklisova‐Murgasova et al. 2012; Kainz et al. 2015; Ebner et al. 2020; Uus et al. 2020), fetal brain MRI atlases (Gholipour et al. 2017; Khan et al. 2019), and automatic segmentation tools (Gholipour et al. 2017; Dou et al. 2021; Karimi et al. 2023; Payette et al. 2023) allow for precise construction and delineation of brain architecture, making it possible to create a true‐scale mechanical growth model. We acquired fetal brain MRIs at two time points for each case: one capturing primary folding patterns and the other depicting developed folding patterns. Unlike other studies, the longitudinal images provided a unique opportunity to assess the performance of the mechanical models in predicting the surface morphology of a developing brain.

We aimed to assess whether a mechanical model can accurately predict the growth and folding trajectory of the fetal brain from an early to a later developmental stage. A key limitation of existing models is the lack of quantitative validation against longitudinal imaging data, which restricts their predictive power. To address this, we used sulcal pits as a validation benchmark. Sulcal pits provide a reliable basis for subject‐specific comparison, unlike more variable markers such as 3‐hinge gyri (Razavi et al. 2021; Zhang et al. 2018; Ge et al. 2018) or gyral peaks (Zhang et al. 2022, 2023). Our success criterion was defined as follows: given a subject's early scan, can the model accurately predict the spatial distribution of sulcal pits at the later time point and correctly identify the matching follow‐up scan? Our results, by meeting the success criterion, showed that for longitudinal scans matched based on sulcal pit similarity, the mechanical model built from the first time point scan was able to generate folded morphologies that closely resembled the actual brain surface at the second time point and accurately identify the corresponding scan, even when the number of sulcal pits did not exactly match.

This study advances the field by providing a quantifiable, data‐driven framework for predictive brain folding simulations, enabling rigorous hypothesis testing in brain development mechanics. It also deepens our understanding of the spatiotemporal evolution of sulcal pits, which is essential for uncovering the processes underlying neurodevelopmental disorders and the factors driving both variability and consistency in human cortical folding.

## 2 Methods

The data for this study were collected from fetal MRI scans of pregnant women who underwent research fetal MRIs at Boston Children's Hospital. The study was approved by the institutional review board committee, and written informed consent was obtained from all participants. All images were acquired with 3‐Tesla Siemens Skyra, Trio, or Prisma scanners using 18‐ or 30‐channel body matrix coils via repeated T_2_‐weighted half‐Fourier acquisition single‐shot fast spin echo scans in prescribed orthogonal planes of the fetal brain. The slice thickness was 2 mm with no inter‐slice gap, in‐plane resolution was between 0.9 and 1.1, and acquisition matrix size was 256 × 204, 256 × 256, or 320 × 320, which were adjusted based on the size of the maternal body and other factors such as the position of the mother inside the scanner and the position of the fetus. Volumetric images were reconstructed using one of the iterative slice‐to‐volume reconstruction algorithms (Kuklisova‐Murgasova et al. 2012; Kainz et al. 2015; Ebner et al. 2020) that generated the highest quality reconstruction based on visual inspection. For this purpose, several reconstructions from the reconstruction algorithms obtained from using various combinations of the original scans were visualized in ITKSNAP, and the images were assessed and compared based on the sharpness and contrast of the reconstructions as well as the presence of any residual motion artifacts. This was done to ensure that the best quality reconstructions were obtained for the downstream tasks that included segmentation and surface analysis. Subsequently, the fetal brain was extracted and registered to a standard atlas space in a procedure described in Gholipour et al. (Gholipour et al. 2017). The resulting 3D images had isotropic voxel size of 0.8 mm.

In this study, we used super‐resolution reconstructed structural (T_2_‐weighted) MRI scans of 24 fetuses, each scanned at two different time points: the first time corresponding to early gestational weeks (GW) (26 to 31 weeks, referred to as the early stage) and the second capturing later gestational weeks (35 to 39 weeks, referred to as the later stage). A detailed overview of the cases and their gestational ages is presented in Table 1.

**TABLE 1:** Gestational weeks (GW) of two time‐point MRI scans from 24 cases.

Case number	GW of scan 1	GW of scan 2	Case number	GW of scan 1	GW of scan 2
1	30.14	38.14	13	26.71	38
2	27.14	36.86	14	29.86	38.71
3	29.43	37	15	27.86	35.86
4	26.71	38.71	16	29.71	36.29
5	30	37.86	17	27.71	36
6	28.14	37.14	18	28.75	37
7	28.86	36.29	19	29.57	39.57
8	29.57	35.57	20	29.43	39.57
9	31.86	35.57	21	27.71	36.43
10	26.78	35.47	22	28.71	36.71
11	28	33	23	29	35.57
12	28	38	24	27.71	34

### 2.1 Reconstruction of White Matter and Pial Surfaces of Early‐Stage Scans

**FIGURE 1:**
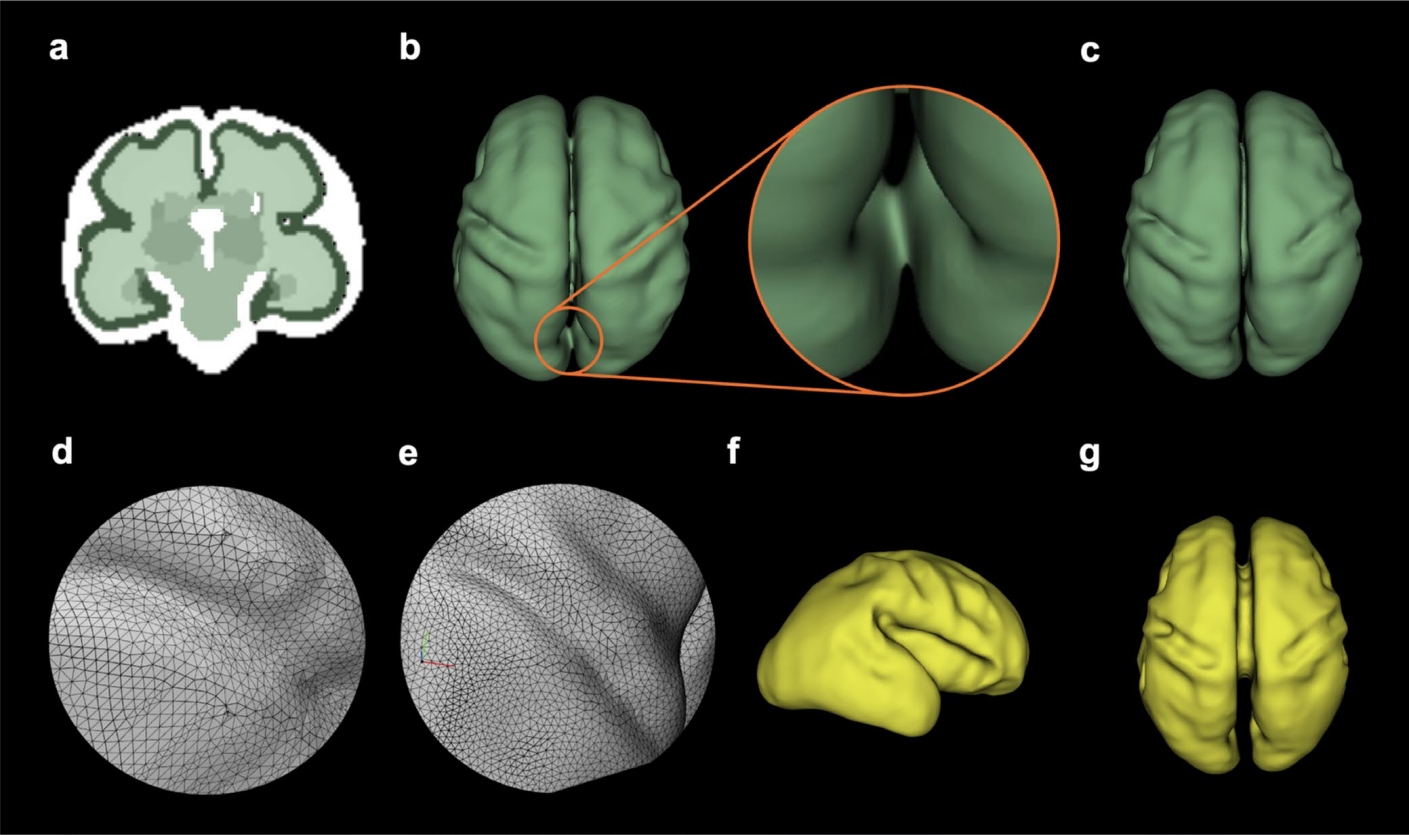
Reconstruction of pial and white matter surfaces. (a) MRI image of a case at 27 gestational weeks with automatic tissue segmentations overlaid on the anatomy (in this study we only used the white matter‐cortical gray matter surfaces and did not use the other segmented structures). (b) Pial surface from automatic gray matter segmentation without manual modification, showing a close‐up of a structural error. (c) Pial surface after manual modification. (d) Irregular mesh of the surface. (e) Surface after smoothing and uniform remeshing, (f–g) Sagittal and axial views of the white matter surface.

Extracting cortical and white matter surfaces from MRI data is a critical step in constructing accurate finite element method (FEM) models of the entire brain. To develop a true‐scale mechanical model for brain growth and folding, we reconstructed the initial smooth state of fetal brain scans obtained during the early stages of development (first time point scans) (Figure 1a). We used a combination of atlas‐based segmentation and deep learning (Gholipour et al. 2017; Dou et al. 2021) to segment the super‐resolution reconstructed fetal MRI scans to different tissue types including the developing white matter and the cortical plate (the cortical gray matter). In specific, an atlas‐based segmentation method designed and validated previously (Gholipour et al. 2017) was used to segment various structures including the developing white matter, cortical plate, cerebrospinal fluid (CSF), and deep gray matter structures. This technique used (1) ANTS diffeomorphic deformable registration to register three atlases in one‐week intervals of the query subject's age to the subject anatomy, and (2) probabilistic STAPLE (Akhondi‐Asl and Warfield 2013) for label fusion among the three propagated label maps obtained from the registrations in step 1. For finer cortical plate segmentation, a fully supervised deep attentive convolutional neural network with U‐shaped skip connections throughout the backbone network and stage‐wise attention refinement modules were used. For the details of the model, training, and validation of this network, we refer to Dou et al. (Dou et al. 2021) The cortical plate segmentations obtained from this network propagated on top of the segmentations obtained from the multi‐atlas segmentation. This helped close some of the holes in the cortical surface.

Multi‐label segmentation and the combination of the multi‐atlas segmentation and the deep learning model led to relatively reliable tissue segmentations. Segmentations for all cases were performed automatically using a consistent pipeline, as described before. For cortical morphological analysis in this study, we only needed segmentations of the developing white matter and the cortical plate; therefore, we did not use the deep gray matter segmentations. Although this approach produced reliable cortical and white matter segmentations, small topological defects sometimes appeared due to imaging artifacts or anatomical complexity (Figure 1b). These included holes, thin bridges, or disconnected surface fragments. Such imperfections are visually noticeable and can also be found in widely used processing pipelines such as FreeSurfer (Fischl 2012; Fischl et al. 1999). To ensure anatomical accuracy and preserve mesh integrity, we systematically corrected these defects using 3D Slicer (Figure 1c). Each surface was carefully examined in both 3D and orthogonal slice views, and localized corrections were applied to maintain continuous and smooth boundaries consistent with known fetal brain morphology. The manual correction protocol was relatively simple and reproducible: any holes in the surface were filled using the paint tool in 3D Slicer with a brush radius set to 1 voxel, and spurious connections between the brain hemispheres and across the cortical plate were removed using the erase tool with the same brush radius. After these minor corrections, Taubin smoothing was applied to the surfaces to preserve overall geometry while improving mesh quality (discussed in detail later). All manual corrections and smoothing were performed by the same trained researchers to ensure consistency. These corrections were performed uniformly across all subjects and were essential for creating topologically accurate meshes suitable for FEM simulations. The entire inspection, editing, and smoothing process typically required between 30 min to 1 h per subject/image, with careful review in both 2D and 3D visualization modes within 3D Slicer.

The cortical surface extraction process produced an irregular triangular mesh containing approximately 30 k vertices (Figure 1d). The 2D meshes are used solely to reconstruct the gray and white matter surfaces, which will later be enclosed and meshed with 3D elements for use in the finite element model. To enhance the mesh's uniformity and optimize it for further analyses, we applied Taubin smoothing techniques *using 3D Slicer's Surface Toolbox*. This method improved the signal‐to‐noise ratio by reducing irregularities that arose during the surface reconstruction from MR images (Dahnke and Gaser 2018). Following smoothing, we performed uniform isotropic remeshing, with a target triangle size of 0.4 mm (Figure 1e), resulting in refined meshes that serve as the foundation for subsequent quantitative surface measurements. The precision of these results depends heavily on the mesh quality. Through this refinement, we aimed to create a more reliable and consistent cortical surface representation, ensuring an optimal mesh for robust FEM simulations of the brain.

The selection of smoothing parameters was guided by the methodology of Demirci and Holland (Demirci and Holland 2022), who extensively evaluated cortical surface mesh smoothing strategies and recommended 50 iterations of Laplacian smoothing and 100 iterations of Taubin smoothing as optimal. In our study, we adopted their approach but ultimately used only Taubin smoothing to preserve surface geometry while improving mesh quality. As shown in Figure S1, we visually inspected the effects of 10, 50, and 100 iterations of Taubin smoothing using 3D Slicer's Surface Toolbox and found that 100 iterations yielded smoother and anatomically consistent surfaces without distorting sulcal morphology. Following smoothing, we applied *uniform isotropic remeshing* with a *target triangle size of 0.4 mm*, resulting in refined meshes suitable for surface analysis and FEM simulations.

To generate the white matter surface from the gray matter surface, we applied a uniform inward offset of 1.4 mm, approximating early cortical thickness (Figure 1f–1g). This approach was adopted to ensure a smooth and continuous gray–white matter interface suitable for finite element meshing. In our preliminary efforts, we attempted to extract the white matter surface separately from segmented MRI volumes to better capture regional variations in cortical thickness. However, this method frequently led to mismatched nodes, topological discontinuities, and mesh self‐intersections, particularly in regions of high curvature. These issues resulted in mesh generation failures or distorted elements, leading to instability during FEM simulations.

By contrast, the inward offset method produced a stable geometry without topological issues or self‐intersections, as confirmed by visual inspection. While cortical thickness does vary spatially during early development, the assumed value of 1.4 mm lies within the range of reported physiological values (Xue et al. 2007; Zhang et al. 2016). Furthermore, prior computational modeling studies of cortical folding have demonstrated that while cortical thickness can influence folding wavelengths, assuming an initially uniform cortical thickness does not substantially affect the overall folding pattern or the spatial distribution of primary sulci (Razavi, Zhang, Li, et al. 2015; Tallinen et al. 2014, 2016; Bayly et al. 2013). Thus, this simplification allowed us to balance anatomical plausibility with computational feasibility while focusing on the localization of sulcal pits rather than detailed fold amplitudes.

### 2.2 Reconstruction of White Matter Surfaces of Later‐Stage Scans

**FIGURE 2:**
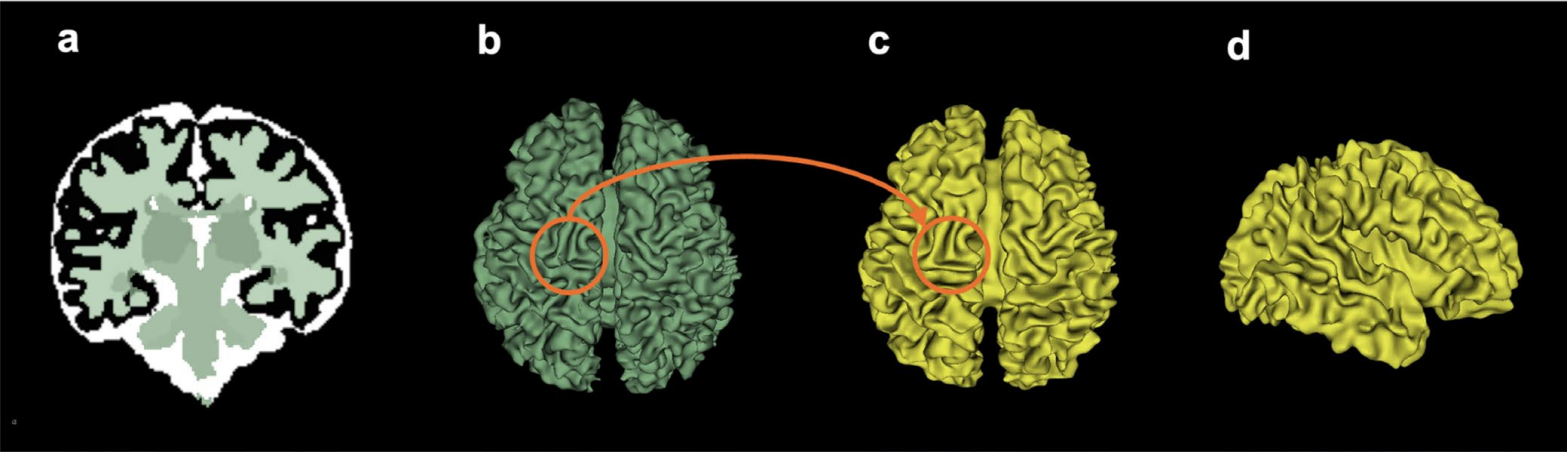
Reconstruction of the white matter surface from the second time point (later‐stage scan). (a) Automatic segmentation of the original MRI image at 38 gestational weeks, (b) The extracted white matter surface without manual modification, (c, d) Axial and sagittal views of the white matter surface following manual modification, smoothing, and remeshing.

To perform the geometric measurements and extract the sulcal and gyral peaks, we focused on the white matter surfaces corresponding to the second time point, representing the later gestational weeks as outlined in Table 1. The extraction process followed the procedures described in the previous section. Initially, the white matter segmentation map (Figure 2a) was generated automatically; however, the white matter surface derived from this map exhibited several geometric inaccuracies (Figure 2b). To address these issues, manual modifications were made to correct the structural errors. Subsequently, smoothing and remeshing procedures were applied to refine the surface. The resulting map, after manual correction, smoothing, and remeshing, is shown in both axial and sagittal views (Figure 2c–2d). Unlike the early‐stage models, where the white matter surface was generated by offsetting the cortical surface, here we directly extracted the white matter surface from the segmented MRI data. These steps were essential for ensuring that the white matter surfaces were of sufficient quality for accurate geometric measurements and further analysis.

### 2.3 Constitutive Framework

To model the growth and folding of the human brain, we adopt the well‐established DTG mechanism as the primary driver of brain folding (Caviness 1975). In DTG, differential expansion rates between the rapidly growing cortical plate and the slower‐expanding subcortical regions generate compressive forces. These forces create mechanical instability, ultimately leading to the buckling of the cortical plate, which drives the characteristic folding patterns of the brain (Caviness 1975). Since the cortex and white matter exhibit distinct growth behaviors, it is crucial to implement unique constitutive equations for each region to accurately capture their individual growth mechanisms.

#### 2.3.1 Multiplicative Decomposition of the Deformation Gradient

We consider a continuum body, denoted as $B_{R}$, which is defined by the region of space it occupies in a fixed reference configuration. Within this body, arbitrary material points are represented as X. As time progresses, the referential body $B_{R}$ experiences a motion described by $x=\varphi \left(X,t\right)$, which leads to the formation of the current deformed body, denoted as $B_{t}$. The deformation gradient captures the transformation undergone by the body during this process. The symbol ∇ represents the gradient operator applied with respect to the material point X in the reference configuration.

(1) $$F=\nabla_{X}\varphi \left(X,t\right)$$

In the field of continuum mechanics, the noticeable deformation of biological tissue can be interpreted as a combination of two mechanisms: growth, the deformation due to an increase in size or number of cells and cell processes, and elastic deformation, which is caused by mechanical forces (Rodriguez et al. 1994). Then, the total deformation gradient can be expressed as follows

(2) $$F=F^{e}.F^{g}$$

where $F^{e}$ is the elastic deformation gradient tensor and $F^{g}$ is the growth deformation gradient tensor. This decomposition enables us to account for both elastic $F^{e}$ and irreversible growth‐related $F^{g}$ components, which provide a comprehensive understanding of the overall deformation experienced by the body.

#### 2.3.2 Constitutive Model for Cortex

The imaging data indicate that cortical thickness undergoes changes during the early developmental stage (Liu et al. 2021). However, in the later stages, it is primarily the alteration in surface area that initiates cortical folding. These findings suggest that the growth of the cortex primarily occurs tangentially, as supported by several studies (Razavi, Zhang, Li, et al. 2015; Ronan et al. 2014). Therefore, we adopted a model for cortical growth that considers in‐plane area expansion, while it assumes purely elastic behavior in the direction normal to the cortical layer, following the approach proposed by Holland et al. (Holland et al. 2015).

(3) $$F_{\mathrm{ctx}}^{g}=\sqrt{\vartheta_{\mathrm{ctx}}^{g}}I+\left(1-\sqrt{\vartheta_{\mathrm{ctx}}^{g}}\right)n_{0}⊗n_{0}$$

Equation (3) incorporates a growth multiplier $\vartheta_{\mathrm{ctx}}^{g}$, the referential unit normal $n_{0}$ of the pial surface, second order unit tensor I, and accounts for the increase in the cortical area. The growth multiplier $\vartheta_{\mathrm{ctx}}^{g}$ can be expressed as follows

(4) $$\vartheta_{\mathrm{ctx}}^{g}=\det\left(F_{\mathrm{ctx}}^{g}\right)$$

A linear kinetic model for the growth of the cortex (gray matter) was used (Holland et al. 2015).

(5) $${\dot{\vartheta }}_{\mathrm{ctx}}^{g}=G^{\mathrm{ctx}}$$

where $G^{\mathrm{ctx}}$ is the cortical growth rate. Then the elastic deformation gradient can be explicitly calculated by the following equation

(6) $$F_{\mathrm{ctx}}^{e}=F_{\mathrm{ctx}}.F_{\mathrm{ctx}}^{g-1}=\frac{1}{\sqrt{\vartheta_{\mathrm{ctx}}^{g}}}F_{\mathrm{ctx}}+\frac{\sqrt{\vartheta_{\mathrm{ctx}}^{g}}-1}{\sqrt{\vartheta_{\mathrm{ctx}}^{g}}}\left(F_{\mathrm{ctx}}.n_{0}\right)⊗n_{0}$$

Lastly, the elastic left ($b^{e}$) and right ($C^{e}$) Cauchy‐Green tensors can be derived from $F_{\mathrm{ctx}}^{e}$, respectively

(7) $$b^{e}=F_{\mathrm{ctx}}^{e}.F_{\mathrm{ctx}}^{\mathrm{et}}$$

(8) $$C^{e}=F_{\mathrm{ctx}}^{\mathrm{et}}.F_{\mathrm{ctx}}^{e}$$

The left tensor $b^{e}$, defined in the deformed configuration, is often used to compute stress quantities such as the Cauchy stress. In contrast, the right tensor $C^{e}$, defined in the reference configuration, is used to evaluate strain energy and deformation relative to the original state. These tensors are fundamental in nonlinear elasticity and allow us to accurately describe how tissue elements stretch and rotate under large deformations, without relying on small‐strain assumptions (Bonet and Wood 2008).

In summary, the model assumes that the cortex undergoes anisotropic tangential growth, with expansion occurring primarily in the plane of the cortical surface. This choice reflects observations from developmental imaging studies showing that cortical folding is driven predominantly by surface area expansion rather than thickness increase in later gestational stages (Huang et al. 2022). The growth‐induced mismatch between the faster‐expanding cortex and the slower‐growing white matter generates compressive stresses in the tangential direction, which drive buckling and the emergence of folds. This mechanism aligns with the differential tangential growth hypothesis and allows for the simulation of realistic folding patterns.

#### 2.3.3 Constitutive Model for White Matter

During the developmental process, there is an observed increase in the volume of white matter (Holland et al. 2015). Therefore, we consider that the growth of the white matter is isotropic. The growth tensor is

(9) $$F_{\mathrm{sub}}^{g}={\vartheta_{\mathrm{sub}}^{g}}^{1/3}I$$

where I denotes the second order unit tensor and $\vartheta_{\mathrm{sub}}^{g}$ is the growth parameter represents the increase in the volume of the white matter

(10) $$\vartheta_{\mathrm{sub}}^{g}=\det\;\left(F_{\mathrm{sub}}^{g}\right)=J^{g}$$

The growth rate of the white matter is defined as follows:

(11) $${\dot{\vartheta }}_{\mathrm{sub}}^{g}=G^{\mathrm{sub}}$$

Similar to the previous brain folding models (Tallinen et al. 2014; Razavi, Zhang, Liu, and Wang 2015), we used a standard neo‐Hookean hyperelastic material model with the depicted free energy function in Equation (12) for the white matter and the cortex.

(12) $$W=\frac{\mu }{2}\left({J^{e}}^{-\frac{2}{3}}\mathrm{tr}\left(F_{\mathrm{sub}}^{\mathrm{et}}.F_{\mathrm{sub}}^{e}\right)-3\right)+\frac{k}{2}{\left(J^{e}-1\right)}^{2}$$

where μ is the shear modulus, k is the bulk modulus, and $J^{e}≔\det\left(F_{\mathrm{sub}}^{e}\right)>0$ is the Jacobian. We introduce the Cauchy stress tensor of the white matter generated by the elastic deformation tensor in the material configuration as follows

(13) $$T={J^{e}}^{-1}F_{\mathrm{sub}}^{e}.\frac{\partial W}{\partial F_{\mathrm{sub}}^{\mathrm{eT}}}$$

where $J^{e}$ is the elastic volume change ratio.

In summary, unlike the cortex, the white matter is modeled to undergo isotropic volumetric growth, expanding uniformly in all directions. This simplification is motivated by the relatively homogeneous and radially symmetric nature of early white matter development. Isotropic growth contributes to the overall increase in brain volume and provides the mechanical foundation upon which the tangentially expanding cortex can fold. This growth framework enables the generation of realistic cortical folding patterns driven by the differential growth between the faster‐growing cortex and the more slowly expanding white matter.

### 2.4 Computational Model

The simulation geometry is based on T_2_‐weighted, motion‐corrected MRI scans of fetal brains at the early stage of development, as detailed in Table 1. Using the methods described in previous sections, we constructed the cortical layer and white matter from the MRI data. The two volumes were merged and uniformly meshed with a mesh size of 0.4 mm, resulting in approximately 7 million tetrahedrons. The gray–white matter boundary was preserved using a conforming tetrahedral mesh, where elements on both sides of the interface shared common faces and nodes, ensuring mechanical continuity across the cortex–subcortex interface. This meshing ensured a minimum of four tetrahedral layers across the cortical thickness. The constitutive models described earlier were implemented in the Abaqus/Explicit finite element package via a user‐defined material subroutine (VUMAT). We modeled the brain as a neo‐Hookean material with the strain energy density defined by Equation (12). The majority of experimental evidence supports that the cortex is somewhat stiffer than the white matter in the mature human brain (Budday et al. 2017, 2019; Hou et al. 2025), although reported ratios can be an order of magnitude apart depending on sample preparation, post‐mortem interval, and testing conditions (Budday, Nay, et al. 2015; Miller 2011). To align with similar studies (Tallinen et al. 2014, 2016), we considered the same shear modulus for both the cortex and white matter (Tallinen et al. 2016). According to Tallinen et al. (Tallinen et al. 2014), a uniform pressure of 0.7 *μ* (where *μ* is the shear modulus of gray matter) was applied to simulate the mechanical constraint imposed by the meninges and developing skull, contributing to the characteristic flattening of cortical folds.

To determine a biologically plausible growth mismatch, we analyzed MRI data from 24 fetal subjects scanned at two developmental stages between 26 and 39 gestational weeks (Table 1). For each case, we measured cortical surface area and white matter volume, estimating the growth ratio $G_{\mathrm{ctx}}/G_{\mathrm{sub}}$ under a linear growth assumption. The resulting ratios ranged from 1.55 to 5.22, and we selected a representative value of 4 for our simulations, which lies near the center of this range. Although we used a fixed $G_{\mathrm{ctx}}/G_{\mathrm{sub}}$, each brain model was grown until its volume approximately matched that of the brain at the second time point. Figure S2 shows that the simulated cortical surface areas and white matter volumes closely match the MRI data at later developmental stages. Under this growth scenario, the corresponding linear stretch ratio (~1.3) is consistent with values used in previous folding models (Tallinen et al. 2014).

### 2.5 Sulcal Pits Extraction and Analysis

Here, we delineate the process for analyzing sulcal pits, which includes brain registration, sulcal pit extraction, and the matching of sulcal pits between two brains. The similarity of sulcal pits from the two brains is computed and subsequently analyzed.

#### 2.5.1 Sulcal Pits Extraction

**FIGURE 3:**
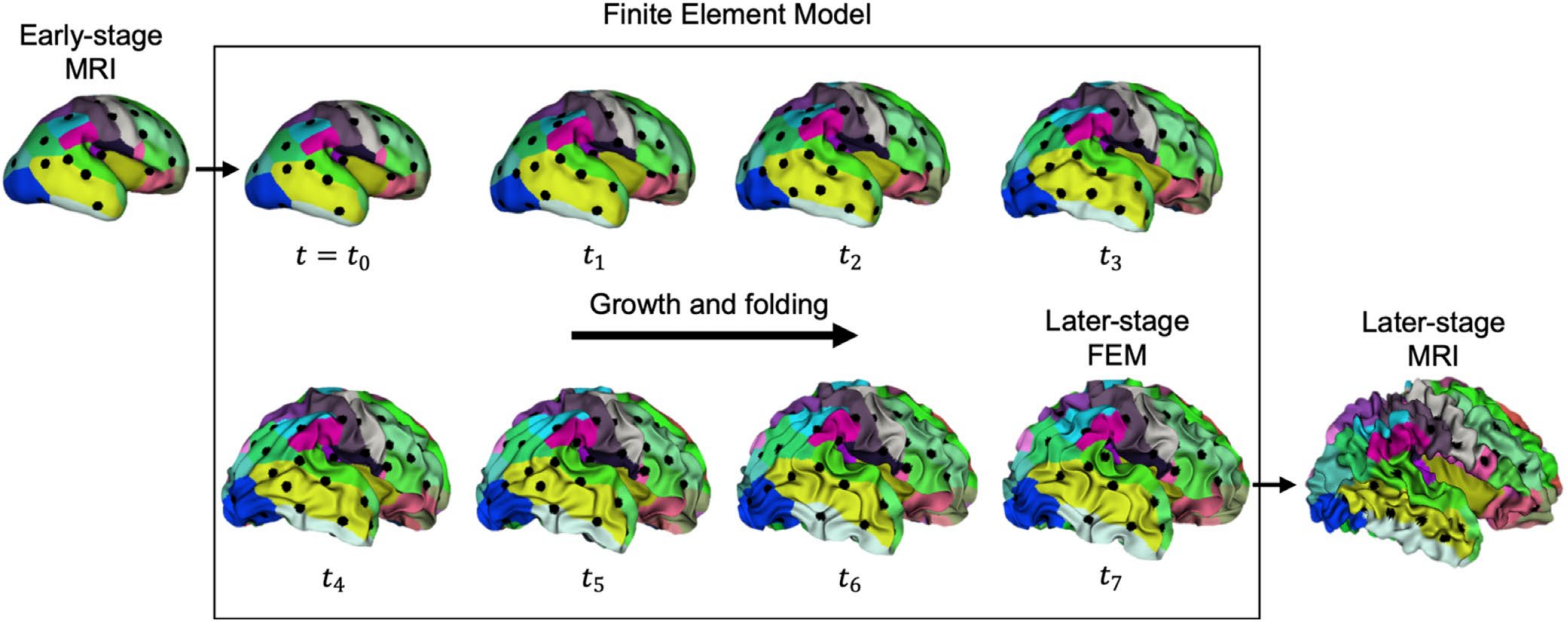
Extracted sulcal pits overlaid on the corresponding brain white matter surface partition map for various developmental stages. The left panel displays sulcal pits from the early‐stage MRI, while the middle panel shows the brain model at the initial stage (*t*_0_), serving as the input for the FEM model, along with the FEM‐simulated brain models at seven developmental stages (*t*_1_ to *t*_7_). The right panel presents the later‐stage MRI. Note that the brain model at the initial stage is identical to that from the early‐stage MRI. Different colors represent distinct cortical regions, with the detailed color coding provided in Figure S3.

Sulcal pits are defined as the deepest points of the sulcal basins of the cerebral cortex (Lohmann et al. 2008; Im et al. 2010). We extracted the sulcal pits automatically according to the pipeline described in Auzias et al. (Auzias et al. 2015). The extraction process primarily involves two steps: estimating a sulcal depth map and extracting the pits using a watershed‐by‐flooding algorithm (Rettmann et al. 2002), while filtering out noisy sulcal pits. The Depth Potential Function (DPF) introduced in Boucher et al. (Boucher et al. 2009) was used to estimate the sulcal depth map. The DPF is reference free, independent of brain size and it has only one parameter α=0.03 that controls the trade‐off between mean curvature and average convexity. After estimating the sulcal depth map using DPF, we applied the watershed‐by‐flooding algorithm to generate sulcal basins and the corresponding sulcal pits. During the watershed flooding, sulcal basins were merged if ridge height (R) was less than Threshold_R = 1.5 and the distance between two pits (D) was less than Threshold_D = 20. At the end of the flooding, any sulcal basin with a basin area (A) less than Threshold_A = 50 was further merged with its neighbor that shares the longest border. Figure 3 illustrates the extracted sulcal pits from early‐stage fetal brain MRI, alongside FEM‐simulated brains at seven developmental stages, presented in chronological order. For comparison, the sulcal pits from the later‐stage brain MRI from the same fetus are also included in Figure 3.

#### 2.5.2 Sulcal Pits Matching

The pattern of sulcal pits extracted from each brain was represented as a 3D point cloud. Consequently, the task of matching sulcal pits between two brains was thus formulated as a points‐matching problem involving these two 3D point clouds. To solve this, we employed the Reweighted Random Walk Matching (RRWM) algorithm (Minsu et al. 2010), which was used to perform point cloud matching between two brains by aligning their respective sulcal pits. For each brain, we considered the 3D coordinates $\left[x,y,z\right]$ of the sulcal pits as the geometric features of the points. The goal was to find the optimal correspondence between the sulcal pits of two brains by minimizing the differences in these features. Let $P\in R^{I\times 3}$ and $Q\in R^{J\times 3}$ represent the two sulcal pit point clouds, with I and J being the number of sulcal pits in each brain. We first constructed an affinity matrix $A\in R^{I\times J}$ using a Gaussian kernel, which quantified the similarity between potential correspondences of points in P and Q as presented in Equation (14).

(14) $$A\left[i,j\right]=\exp\left(-\frac{{\left\|P_{i}-Q_{j}\right\|}^{2}}{\sigma }\right)$$

where $P_{i}$ and $Q_{j}$ are the *i*th row of P and *j*th row of Q, i=1,⋯,I,j=1,⋯J. ${\left\|P_{i}-Q_{j}\right\|}^{2}$ is the squared Euclidean distance between $P_{i}$ and $Q_{j}$. *σ* is a scaling parameter that controls the “spread” of the Gaussian function, and it is set to 100.

The RRWM algorithm was then applied to the affinity matrix A, yielding a matching matrix $M\in R^{I\times J}$, where each entry represented the likelihood of correspondence between a pair of sulcal pits from the two 3D point clouds. After discretizing the matching matrix M, we obtained a set of matched sulcal pits, with the number of matches being the minimum of I and J. For each pair of matched sulcal pits, we further calculated a pairwise similarity score using the Gaussian kernel affinity function, resulting in similarity values ranging from 0 to 1. To refine the matches, we applied a similarity threshold of 0.5, eliminating any weakly paired sulcal pit pairs. Only strongly paired sulcal pits are considered as paired sulcal pits.

#### 2.5.3 Brain Registration

Since our primary focus is on matching sulcal pits between two brains of different sizes, such as early‐stage and later‐stage brains, it is important to consider that brain size, which influences the 3D positions $\left[x,y,z\right]$ of the sulcal pits, can affect the accuracy of the point cloud matching algorithm. To enhance the robustness of sulcal pits matching results, we designed a two‐stage position‐based registration: the first stage coarse registration, sulcal pits matching, second stage refined registration, sulcal pits matching again.

The first stage involved a coarse registration by rescaling the early‐stage brain along the positive and negative *x*, *y*, *z* dimensions to align it with the reference brain from the later stage. The coarse registration step works well because the origins of both brains are located at the same anatomical position. This rescaling was necessary because the growth rates of the brain differ across these six dimensions. The rescaling factors were determined based on the relative positions of the sulcal pits in corresponding space of the two brains. For instance, let $P_{\mathrm{xp}}\in R^{N\times 3}$ represent *N* sulcal pits located in the positive x‐dimension of the brain to be scaled, and $Q_{\mathrm{xp}}\in R^{M\times 3}$ represent *M* sulcal pits in the positive x‐dimension of the reference brain. The positive x‐factor $f_{\mathrm{xp}}$ was calculated by Equation (15) as follows.

(15) $$f_{\mathrm{xp}}=\frac{\text{mean}\left(P_{\mathrm{xp}}^{1}\right)}{\text{mean}\left(Q_{\mathrm{xp}}^{1}\right)}$$

where $P_{\mathrm{xp}}^{1}\in R^{N\times 1}$ denotes the x‐coordinates of the *N* sulcal pits from the brain being scaled and $Q_{\mathrm{xp}}^{1}\in R^{M\times 1}$ denotes the x‐coordinates of the *M* sulcal pits from the reference brain. From the two brains with a coarse registration, we can identify strongly paired sulcal pits (with similarity scores exceeding 0.5). as described in the Sulcal pits matching section.

**FIGURE 4:**
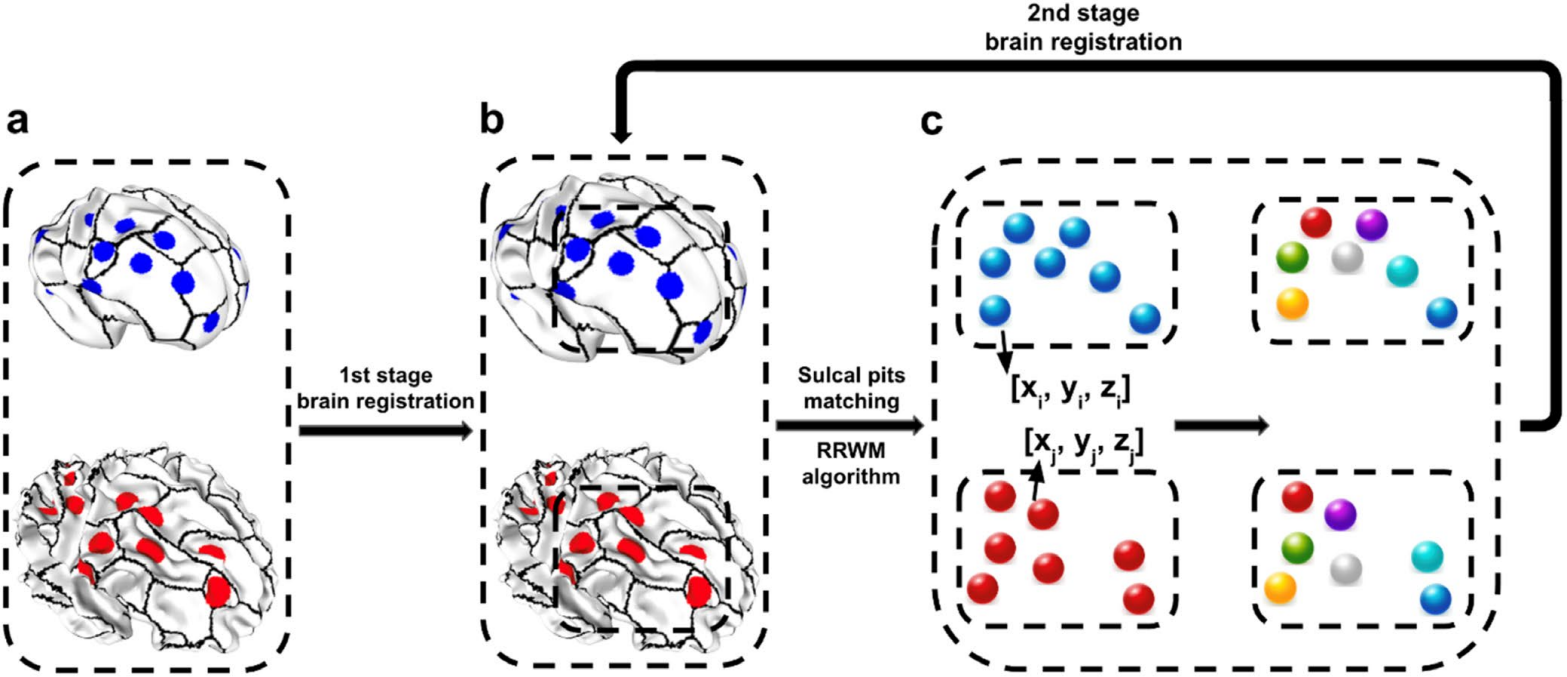
Overview of the sulcal pit matching pipeline. (a) Two brain surfaces with extracted sulcal pits, where pits from two brain are shown in different colors. Black lines delineate the boundaries of sulcal basins. The first stage involves coarse registration to align the two brains to a similar scale, as shown in (b), providing a foundation for subsequent sulcal pit matching. This coarse registration is based on the 3D coordinates of the sulcal pits from both brains. (c) illustrates a subset of these pits as an example. The Reweighted Random Walk Matching (RRWM) algorithm is then applied to identify correspondences between pits, with matched pairs indicated by the same color. Using the subset of matched pits with high similarity scores (greater than 0.5), a second‐stage brain registration is performed to further refine the alignment. Sulcal pit matching is repeated on the refined models to obtain the final matching results.

**FIGURE 5:**
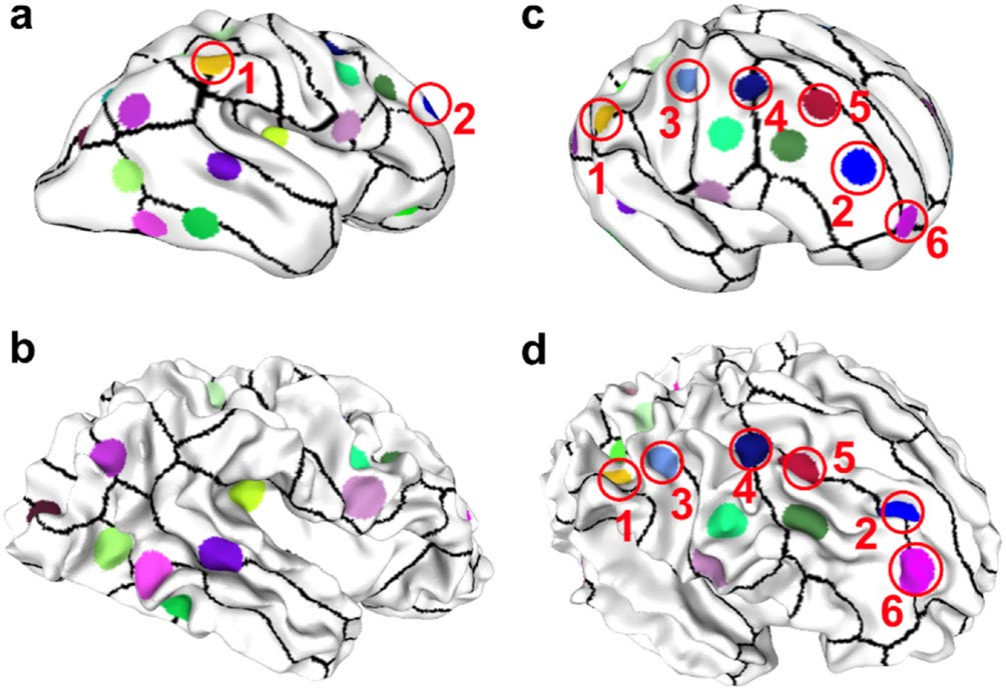
Matching of sulcal pits between early‐stage and later‐stage MRI scans of the same subject. (a) Early‐stage MRI and (b) corresponding later‐stage MRI from the same individual, showing matched sulcal pits. Colored dots represent identified sulcal pits, with matching colors denoting paired pits across the two time points. Black lines delineate the boundaries of cortical parcellations. A very similar spatial distribution was observed for the visible pits across both time points. The yellow (marked “1”) and blue (marked “2”) dots in (a) remained occluded and are not visible in (b). (c, d) display the same matching results of sulcal pits from an alternative viewing angle to improve visualization of the yellow and blue dots. Additional dots become visible from this view angle (marked “3” to “6”). The consistent spatial distribution of these labeled dots across (c) and (d) further supports the robustness of the matching algorithm.

The second stage involved a refined linear registration (Cashbaugh and Kitts 2018) aiming to determine the affine transformation, including translation and rotation, that optimally aligns the strongly paired sulcal pits identified in the first stage. Sulcal pits matching was then performed based on the linearly registered brains, leading to the final matching outcomes. This two‐stage sulcal pits matching approach significantly enhances the accuracy and reliability of our results. Figure 4 illustrates the two‐stage process for matching sulcal pits and Figure 5 shows an example of sulcal pit matching between a pair of early‐stage and later‐stage MRI scans from the same subject, highlighting the robustness of the matching algorithm.

#### 2.5.4 Similarity Degree of Sulcal Pits (SDSP)

The SDSP between two brains was defined based on the matching results obtained from the two‐stage sulcal pit matching method. For each matched pair of sulcal pits, a pairwise similarity score was computed using their 3D coordinates through a Gaussian kernel affinity function (see Equation 14), resulting in similarity values ranging from 0 to 1. To refine the matches, a similarity threshold of 0.5 was applied to remove weakly paired sulcal pits. Only strongly paired sulcal pits, with similarity scores above this threshold, were retained. The overall SDSP was then calculated as the average similarity across these strongly paired sulcal pits.

### 2.6 Data Analysis

Heatmaps were utilized to illustrate the SDSP between early‐stage MRI and later‐stage MRI, between later‐stage FEM models and later‐stage MRI, and between early‐stage and later‐stage FEM models. In each heatmap, the diagonal values represent the similarity within the same subject, while the off‐diagonal values reflect similarity between different subjects. Based on the stability of sulcal pits within individual brains, we hypothesized that the diagonal SDSP value would be the largest on the same row. In other words, the distribution of sulcal pits at the early stage should be more similar to those at the later stage of the same subject, rather than to those of other subjects. To examine the evolution of sulcal pits during brain development, we used FEM to simulate seven developmental stages (*t*_1_, *t*_2_, …, *t*_7_) starting from the early‐stage MRI model (*t*_0_). We then calculated the SDSP values between these developmental stages and *t*_0_. Paired *t*‐tests were conducted to compare the SDSP values between two adjacent developmental stages and the early stage, specifically comparing the SDSP values between *t*_*i*_ and *t*_0_ versus those between *t*_*i−*‐1_ and *t*_0_ (*i* = 1, …, 7). These *t*‐tests aimed to assess whether there were statistically significant differences in the similarity of sulcal pits between adjacent stages relative to the early stage.

## 3 Results

In this study, we developed a true‐scale, image‐based mechanical model to investigate the mechanics of brain development and folding, with a focus on the spatiotemporal evolution of sulcal pits (deep sulcal roots). Using our growth framework, we applied the model to 24 fetal brain scans at the early‐stage time point and simulated the folding process up to the later‐stage time point. Before developing the mechanical model, the longitudinal MRI data were analyzed to assess how many later‐stage scans could be accurately associated with their corresponding early‐stage scans. Specifically, the goal was to determine the number of individual cases with two MRI scans that exhibit a greater SDSP (similarity degree of sulcal pits) between the early‐stage and later‐stage time points from the same individuals compared to SDSP from different individuals. This step was crucial for selecting cases as ground truth for developing mechanical models, ensuring that the similarity metric demonstrated a stronger correlation between the two stages. In this section, quantified results from the simulations and the MRI data analysis are presented.

### 3.1 Analysis of Longitudinal MRI Data

**FIGURE 6:**
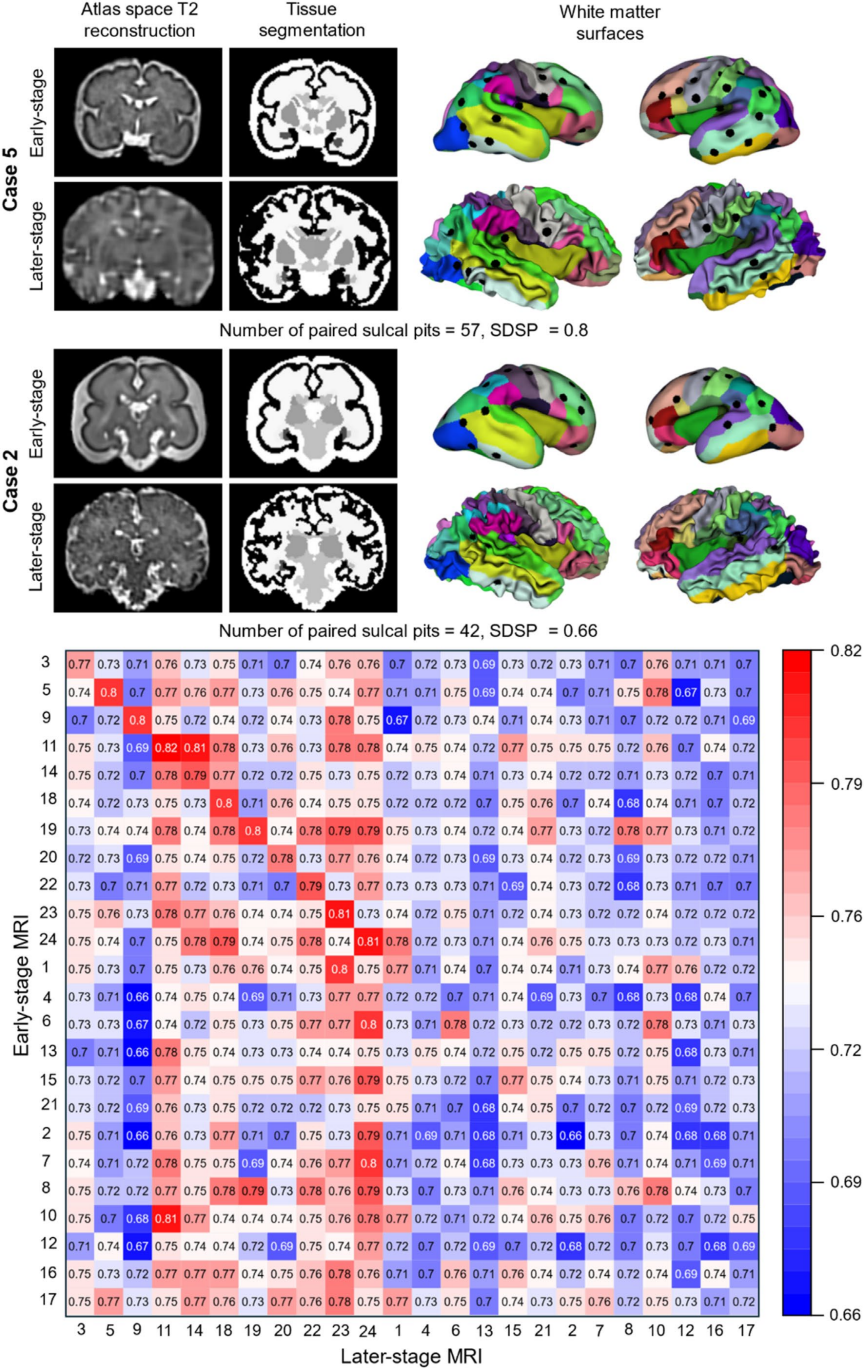
Heatmap showing the similarity degree of sulcal pits (SDSP) between early‐stage and later‐stage MRI scans across all subject pairs. The diagonal elements represent intra‐subject similarity, while the off‐diagonal elements indicate inter‐subject similarity. Due to variability in MRI scan quality, only the first 11 subjects exhibit consistently higher similarity with their own later‐stage scans and are therefore considered reliable cases in the ground truth dataset. This figure also includes coronal views of the T_2_‐weighted MR images in atlas space and the corresponding tissue segmentations for Cases 2 and 5. Full orthogonal MRI views (coronal, axial, and sagittal) for these cases are provided in Figure S4. Different colors correspond to distinct cortical regions, with the full color legend provided in Figure S3.

A total of 24 fetal brains, each with two scans, one at an early stage and another at a later stage of development, were analyzed (Table 1). Figure 6 illustrates the SDSP for all 24 cases, evaluating the similarities between their early‐ and later‐stage MRI scans. We expected the SDSP between two stages of the same fetus to be higher than that between different fetuses. The diagonal SDSP values in Figure 6 represent the similarity between the early‐stage scan and later‐stage scan from the same fetus. In contrast, the off‐diagonal SDSP values in each row indicate the similarity between the early‐stage scan of each case and the later‐stage scans of other cases. When the diagonal value is the highest in each row, it suggests that the later‐stage scan can be successfully matched with the early‐stage scan of the same fetus using our developed sulcal pits similarity algorithm. However, in instances where the diagonal values are not the largest, the algorithm matches the early‐stage scan of one case with the later‐stage scan of an unrelated case.

The algorithm successfully identified 11 cases (sorted and shown as the first 11 rows in Figure 6) that exhibited greater similarity between their own early and later‐stage scans compared to the scans of other cases. This suggests stability in the formation of primary sulcal pits up to the second time point. In contrast, the remaining 13 cases (shown as the last 13 rows in Figure 6) showed lower similarity between their own longitudinal scans than between their early‐stage scans with the later‐stage scans of other cases. It is important to clarify that the heatmap appears scattered because only the diagonal entries (matched scans) are grouped; off‐diagonal SDSP values represent mismatched subject pairs and are not sorted. As such, adjacent cells do not reflect continuous relationships, and the apparent variability arises from natural inter‐subject differences rather than noise. To understand the source of this inconsistency, we investigated potential factors such as the effect of time gaps between two longitudinal scans.

**FIGURE 7:**
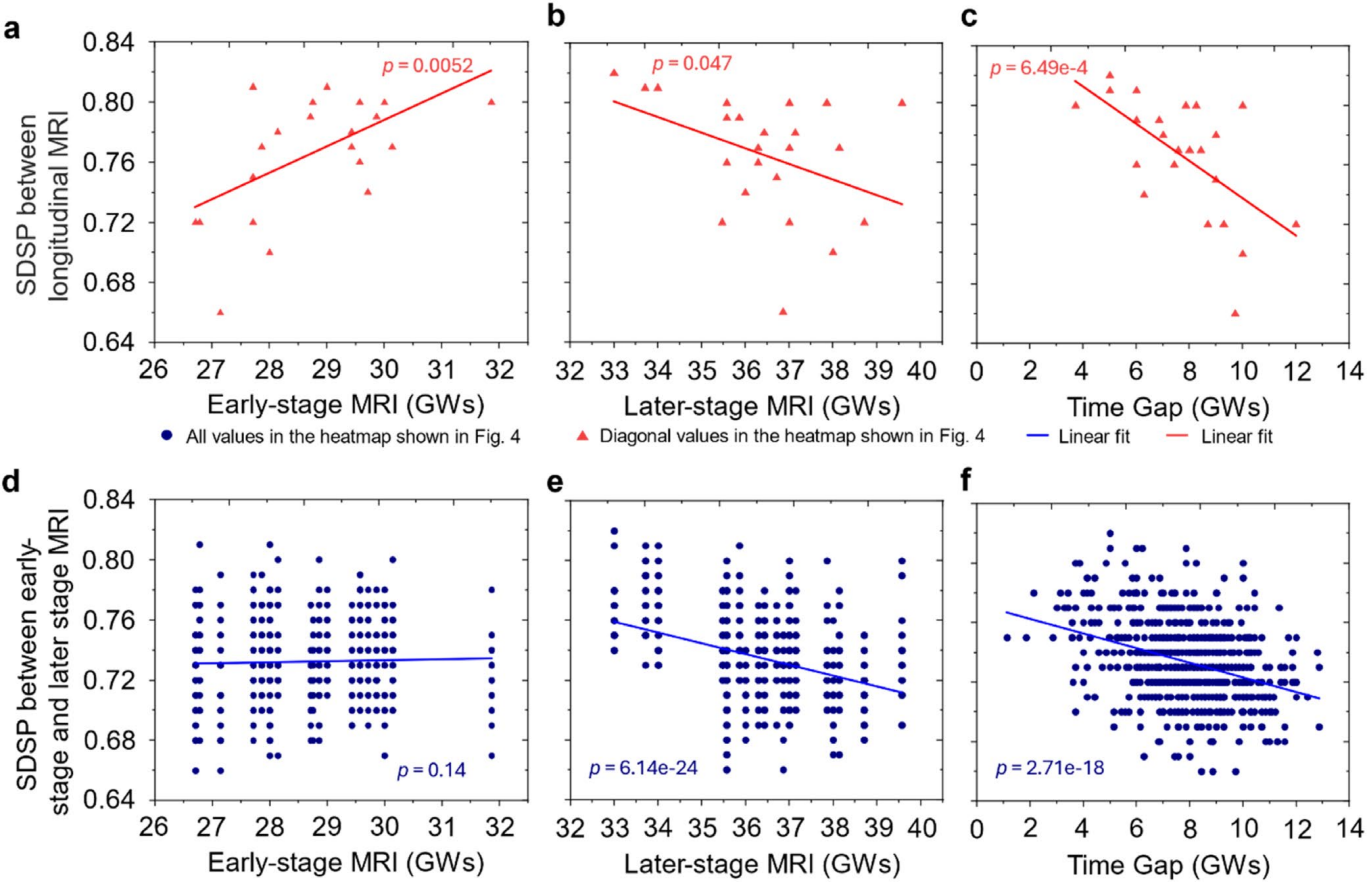
The dependency of the similarity degree of sulcal pits (SDSP) between early‐stage and later‐stage longitudinal MRI scans on the timing of MRI acquisitions and the time gap between scans. (a) The SDSP between early‐stage and later‐stage MRI scans is positively correlated with the gestational weeks at the time of the early‐stage MRI for diagonal cases (*p* = 0.0052), and (d) not significantly correlated for off‐diagonal values (*p* = 0.14). (b, e) The SDSP is negatively associated with later‐stage gestational weeks for both diagonal (*p* = 0.047) and off‐diagonal values (*p* < 10^−6^). (c, f) The SDSP is negatively associated with time gaps between scans for both diagonal (*p* = 6.49 × 10^−4^) and off‐diagonal values (*p* < 10^−6^). Larger time gaps between longitudinal scans are associated with a decrease in SDSP between early‐stage and later‐stage MRI scans.

Noting that the early‐stage and later‐stage scans were not all acquired at the same gestational weeks (see Table 1), we examined the diagonal and off‐diagonal SDSP values of longitudinal scans in relation to the early‐stage gestational weeks, later‐stage gestational weeks, and the time gap between longitudinal scans (Figure 7). The SDSP between early‐stage and later‐stage MRI scans was positively associated with early‐stage (first time point) gestational weeks for diagonal cases (Figure 7a, *p* = 0.0052) but was not significant for off‐diagonal and diagonal values (Figure 7d, *p* = 0.14). It was negatively associated with later‐stage (second time point) gestational weeks for both values (Figure 7b, *p* = 0.047 and Figure 7e, *p* < 10^−6^, respectively) and negatively associated with time gaps for both values as well (Figure 7c, *p* = 6.49 × 10^−4^ and Figure 7f, *p* < 10^−6^, respectively). These relationships are expected, as cortical folding progresses and sulcal pits deepen over time. As a result, the SDSP is smaller with earlier stages of brain development and larger with later stages. The variation in MRI scan times may contribute to the mismatch between the early‐stage and later‐stage scans from different cases for the last 13 fetuses in Figure 6. For instance, compared to the early‐stage scan of case 4, its own later‐stage scan has a smaller SDSP than the later‐stage scans of cases 23 and 24. This is caused by the smaller GW of case 4 at the early‐stage scan (smaller SDSP, red line in Figure 7a) and smaller GWs of cases 23 and 24 at the later‐stage scans (larger SDSP, blue line in Figure 7b). The fetuses with smaller GWs typically exhibit smoother brain surfaces, characterized by shallower sulcal pits. For smooth brain surfaces of different cases, subtle variations of sulcal pits are expected, and therefore larger SDSP values were observed between the early‐stage scan of case 4 and later‐stage scans of cases 23 and 24. Figure 7d–f also illustrate that the SDSP between the early‐stage MRI of one fetus and the later‐stage MRI of a different fetus is still affected by both the gestational age at the later time point and the time interval between scans. This is a key factor explaining why some off‐diagonal SDSP values (comparing different fetuses) may exceed the corresponding diagonal values, which reflect within‐fetus comparisons. To put together, the timing of these longitudinal MRI scans is a confounding factor in matching them from the same fetuses. Another contributing factor is the limited MRI scan quality of some of the unmatched cases, which impacted the ability to accurately match the later‐stage brain MRI scans to their early‐stage counterparts based on the similarity of their sulcal pits.

### 3.2 Growth and Folding of the Brain Model

**FIGURE 8:**
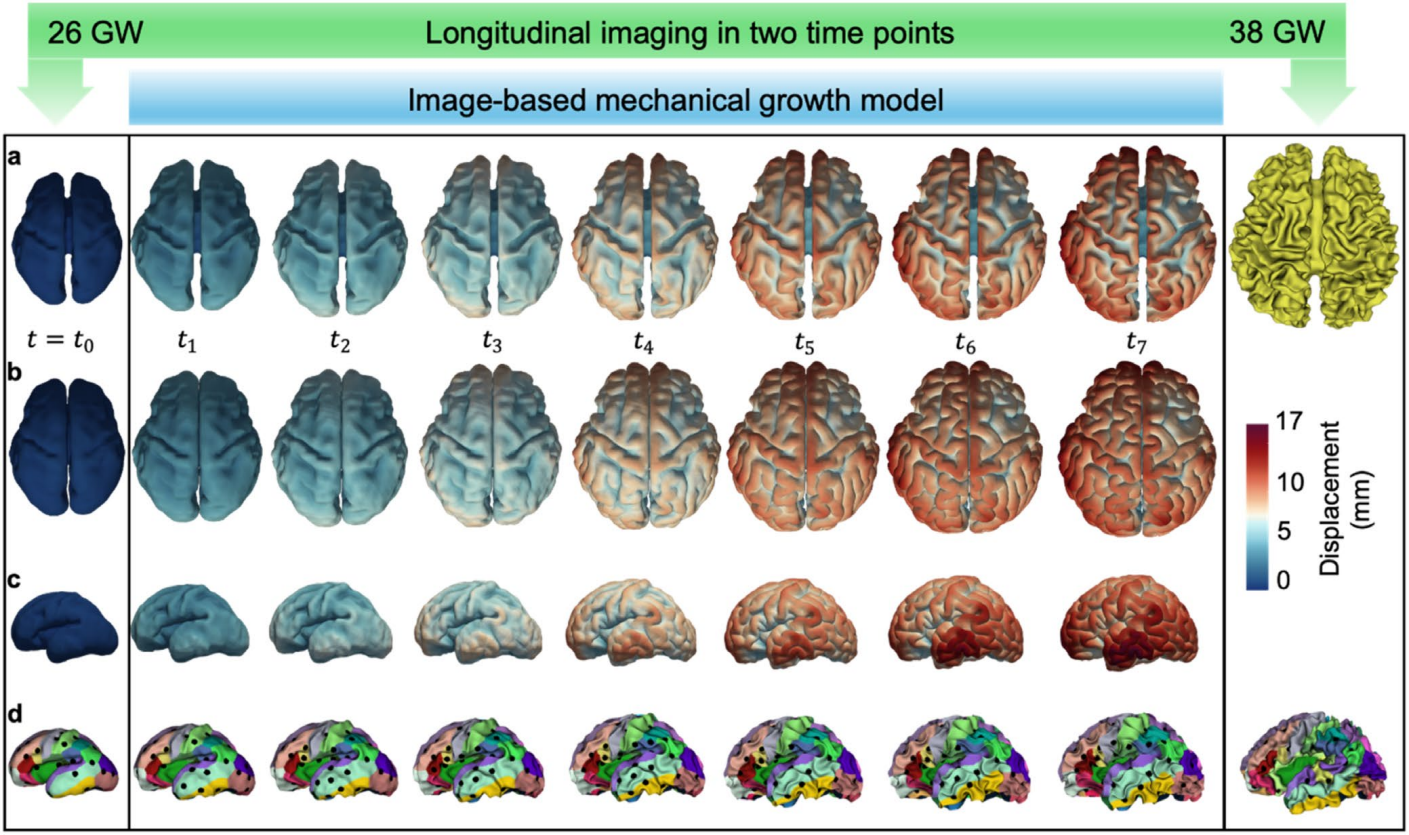
Growth and folding of the simulated brain compared to corresponding MRI‐derived white matter and gray matter surfaces at 26 GW and 38 GW. The simulation starts with a smooth fetal brain at approximately 26 GW and demonstrates the development of gyrification driven by the tangential growth of the cortical layer by 38 GW. (a) Left column: White matter surface from fetal MRI at 26 GW. Middle column: White matter surface of the brain model during growth and folding (*t*_1_ to *t*_7_). Right column: White matter surface from fetal MRI at 38 GW. (b) Left column: Gray matter surface from fetal MRI at 26 GW. Middle column: Gray matter surface of the brain model during growth and folding (*t*_1_ to *t*_7_). Right column: Gray matter surface from fetal MRI at 38 GW. (c) Left column: Side view of the gray matter surface from fetal MRI at 26 GW. Middle column: Side view of the gray matter surface of the brain model during growth and folding (*t*_1_ to *t*_7_). (d) Left column: Sulcal pits from the fetal brain at 26 GW extracted and overlaid on the white matter surface from MRI. Middle column: Sulcal pits of the brain model during growth and folding (*t*_1_ to *t*_7_). Right column: Sulcal pits extracted and overlaid on the white matter surface from MRI at 38 GW. The color contour represents displacement (in mm) in the model. Different colors represent distinct cortical regions, with the detailed color coding provided in Figure S3.

Figure 8 illustrates the growth and gyrification of the brain model for an individual with MRI scans taken at two time points. The first time point, at 26 GW, was used to construct the initial mechanical model, which at this stage is smooth and contains only a portion of the primary folds (*t*_0_ = 0). The model then grows according to the growth framework outlined in the Methods section, gradually developing folds over time, up to the second time point at 38 GW. In alignment with the differential tangential growth (DTG) mechanism, the mismatch in growth rates between the faster‐growing cortex (gray matter) and the slower‐expanding white matter (subcortex) generates compressive forces in the cortex, ultimately leading to mechanical instability and brain folding. The model successfully captures the emergence of secondary and tertiary folds from the primary folds, as well as illustrates the spatiotemporal evolution of sulcal pits. The folding morphology and sulcal pit patterns observed in the model at the second time point qualitatively align with the folding patterns and sulcal pits seen in the corresponding MRI scans. Figure 8 presents seven time steps of the model's growth and folding, although the mechanical model is capable of generating numerous time steps for any growth scenario. This ability to simulate multiple stages of growth is a key advantage, as it offers more detailed insights compared to the typically limited number of longitudinal MRI data points (e.g., two or three) available for each individual case. The dynamic growth and folding process of the model is captured in Video S1.

### 3.3 Performance of Mechanical Models and Prediction of Sulcal Pits Evolution

**FIGURE 9:**
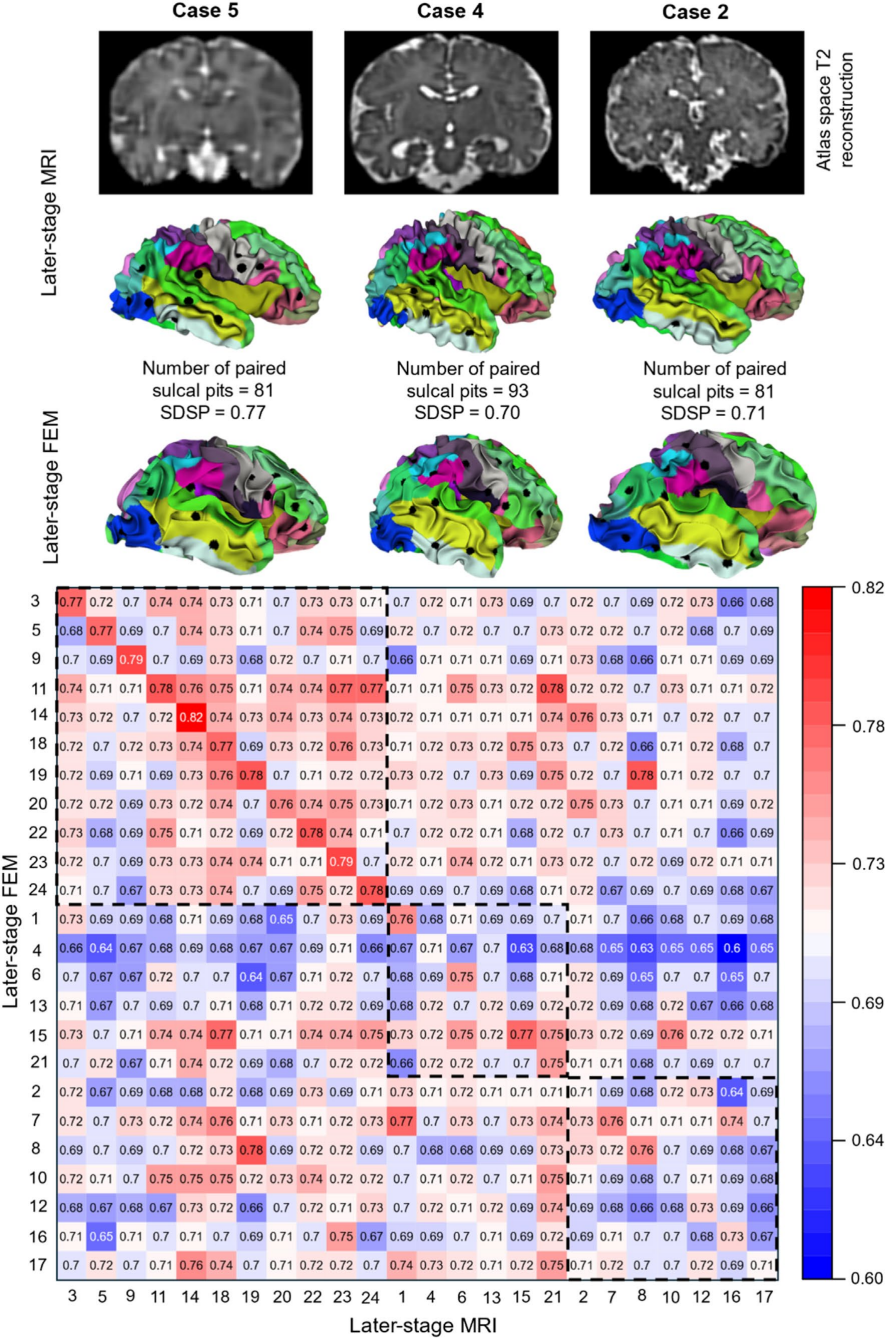
Sulcal pits‐based similarity (SDSP) between later‐stage FEM models and later‐stage MRI scans across all subject pairs. Diagonal values indicate within‐subject similarity, while off‐diagonal values represent between‐subject similarity. Rows 1–11 (top‐left dashed box): Cases showing higher SDSP values between the later‐stage FEM model and their corresponding later‐stage MRI scans compared to unrelated cases. Case 5 is presented as a representative example, including extracted sulcal pits from the FEM model and MRI scans, along with a coronal view of the T_2_‐weighted MR image in atlas space. Rows 12–17 (middle dashed box): Cases displaying higher similarity between the later‐stage FEM model and MRI scans from the same fetuses, even though the greatest similarity was not observed between early‐stage and later‐stage MRI scans. Case 4 is shown as an example, including sulcal pits and its MR image. Rows 18–24 (bottom‐right dashed box): Cases where the highest brain similarity is neither between early‐stage and later‐stage MRI scans nor between FEM models and their matching MRI scans. Case 2 is included as a representative example, along with its sulcal pits and MR image. Full orthogonal MRI views (coronal, axial, and sagittal) for these cases are provided in Figure S4. Different colors represent distinct cortical regions, with the full color legend provided in Figure S3.

We simulated the growth and folding models for all 24 cases, including the 13 cases where early‐stage and later‐stage SDSP did not yield the highest values for the same fetuses. Figure 9 presents a heatmap illustrating the similarity of sulcal pits between the later‐stage FEM models and the associated later‐stage MRI scans for these 24 fetal cases. As expected, the 11 cases that exhibited greatest brain similarity between the early‐stage and later‐stage MRI scans from the same fetuses (indicated by the largest value in the diagonal element within each row in Figure 6, rows 1 to 11) also showed highest brain similarity between the later‐stage FEM models and MRI scans for the same fetuses (with the largest diagonal similarity in Figure 9, rows 1 to 11). This result demonstrates the strong predictive performance of the mechanical models in capturing the later‐stage brain morphology and sulcal pit patterns when supplied with robust ground‐truth data. Interestingly, six cases did not demonstrate the greatest brain similarity between the early‐stage and later‐stage MRI scans from the same fetuses (see Figure 6, rows 12 to 17) but did show the highest similarity between the later‐stage FEM model and MRI scans from the same fetuses (i.e., largest diagonal similarity in Figure 9 within the same rows, rows 12 to 17). This finding suggests that the later‐stage brain model generated from FEM model shares a more comparable distribution of sulcal pits with the later‐stage brain MRI scan for the same fetus. However, seven cases exhibited the largest brain similarity neither between the early‐stage and later‐stage MRI scans from the same fetuses (as shown in Figure 6, rows 18 to 24) nor between the later‐stage FEM model and MRI scans from the same fetuses (illustrated in Figure 9, rows 18 to 24). The limited quality of these early‐stage brain MRI scans may have hindered the FEM models from achieving a similar distribution of sulcal pits to the later‐stage MRI scans. Nonetheless, even in these seven cases, the numbers of off‐diagonal elements exceeding the diagonal values was significantly smaller (*p* = 0.028) in the heatmap between the later‐stage FEM and MRI scans (5.57 ± 6.27) than the numbers between the early‐stage and later‐stage MRI scans (12.29 ± 7.76). In each of these three scenarios, FEM models successfully simulate the growth and folding of a smooth brain, resulting in a folded brain that preserves the sulcal pits distribution observed in the real‐MRI scans.

**FIGURE 10:**
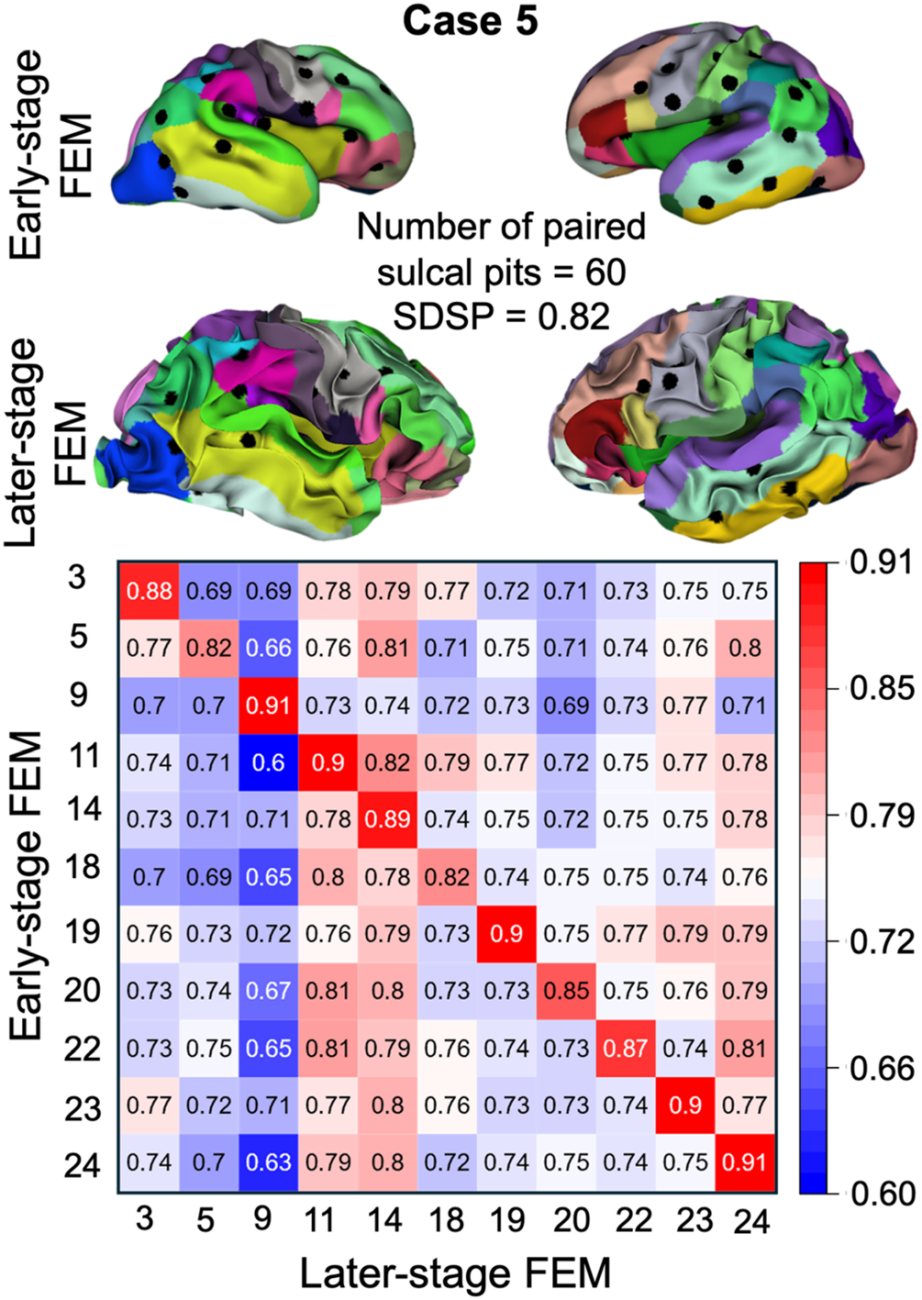
Sulcal pits‐based similarity (SDSP) between the early‐stage and later‐stage FEM models for subjects with higher diagonal values in both ground truth and FEM‐MRI analysis. The diagonal values represent within‐subject similarity, while the off‐diagonal values indicate between‐subject similarity. Case 5, along with its extracted sulcal pits from the early‐ and later‐stage FEM models, is presented as an example. Different colors represent distinct cortical regions, with the detailed color coding provided in Figure S3.

To further evaluate the FEM models' performance, we selected the 11 cases that demonstrated higher sulcal pits similarity between early‐stage and later‐stage MRI scans of the same fetuses, despite the confounding effects of different longitudinal scan times across subjects. For these cases, we generated an SDSP heatmap for both early‐stage and later‐stage FEM models, as shown in Figure 10. In Figure 10, all diagonal values are higher than off‐diagonal values within the same row, demonstrating that the sulcal pits from the later‐stage FEM models (*t*_7_) align with their corresponding early‐stage FEM models and the two FEM models from the same fetuses can be matched with 100% accuracy. This result suggests that the FEM models reliably preserve sulcal pit distribution during brain development. Additionally, the findings suggest that when accurate ground‐truth data at the early stage is available, FEM models can effectively predict the spatiotemporal evolution of sulcal pits. The SDSP heatmap for all FEM models, comparing their early and later stages, is shown in Figure S5. In total, five cases (cases 2, 4, 6, 12, and 13) in Figure S5 were matched to later‐stage FEM models of different cases (cases 11, 24, 24, 24, 24), failing to exhibit a higher SDSP between their associated early‐stage and later‐stage FEM models. A detailed inspection revealed that the initial scan times for three failed cases (cases 2, 4, and 13) had relatively smaller GWs, making the fetal brains appear less similar to their own FEM models (Figure 7). Another observation is that the matched cases (cases 11 and 24) have smaller GWs at the later‐stage scans. These are consistent with the explanation discussed in Figure 7. Therefore, for more accurate prediction of the brain's folded morphology based on its initial state, it is essential that the initial morphology reflects a time when primary folds have already formed on the brain's surface. This also suggests that human brains at early stages of development share greater similarities and, with gradual growth, develop distinct folding patterns and sulcal pits.

### 3.4 Evolution and Establishment of Sulcal Pits

**FIGURE 11:**
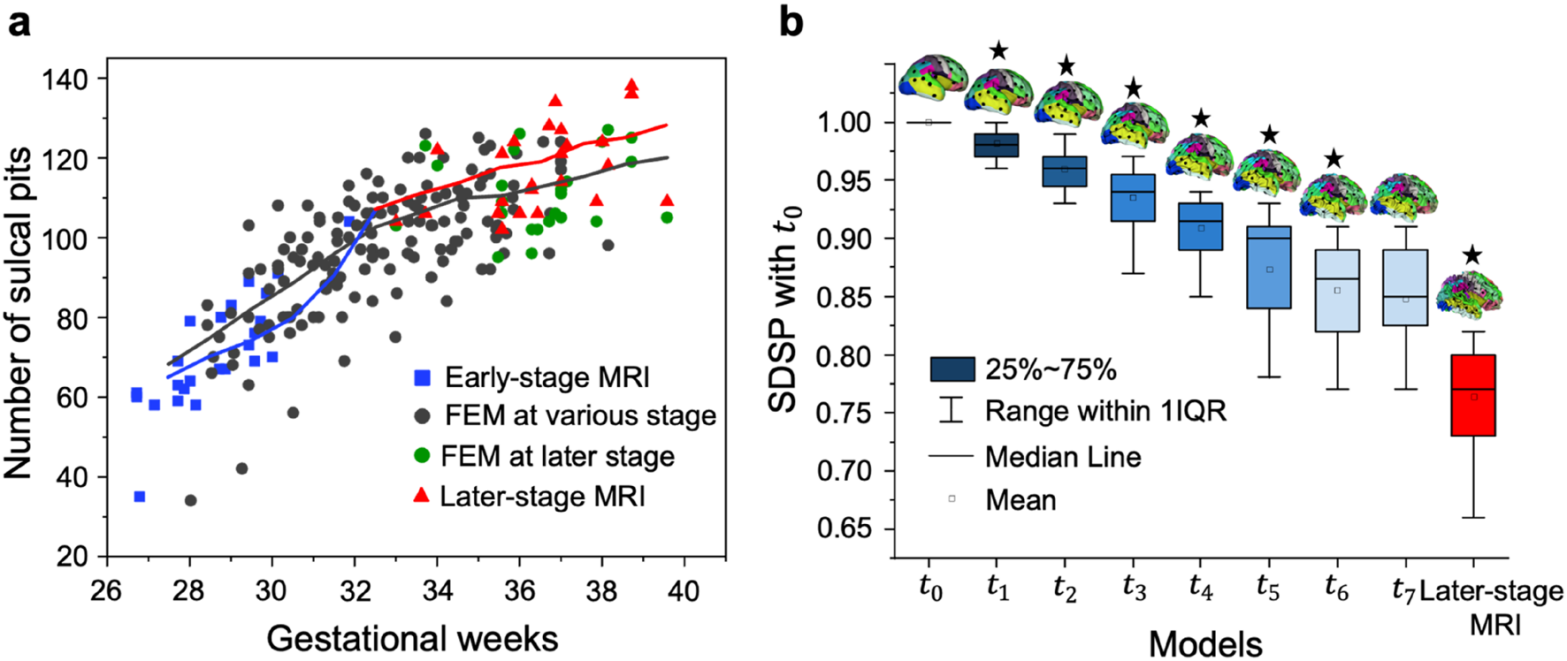
(a) Relationship between gestational weeks and the number of sulcal pits. Squares represent early‐stage MRI, and triangles denote later‐stage MRI, with models from the same subject marked in identical colors. Black dots indicate FEM models sulcal pits across various developmental stages. The black line represents the moving average of FEM models, calculated using a window size of 3 and step size of 1; the blue and red lines show the moving averages of early and later‐stage MRI scans, respectively. Individual trajectories from the FEM models and the MRI models are shown in Figure S7. (b) Similarity degree of sulcal pits (SDSP) between early‐stage MRI and various FEM models' stages/later‐stage MRI. The leftmost bar represents the SDSP of early‐stage MRI with itself (value of 1), blue bars show SDSP between early‐stage MRI and FEM models' stages, and the red bar (rightmost) indicates SDSP between early and later‐stage MRI. Data includes all 24 cases, with error bars indicating standard deviation. Each bar is topped with an example brain model showing sulcal pits (black dots). An asterisk above an example brain model denotes statistically significant difference between two bars at *t*_*i*_ versus *t*_*i*−1_ (*i* = 1, …, 7). The bar at the later‐stage MRI was compared with the bar at *t*_7_. Different colors represent distinct cortical regions, with the detailed color coding provided in Figure S3.

A major challenge in understanding the spatiotemporal evolution of sulcal pits in individual brains is the limited availability of longitudinal data for each case. To examine how FEM models can address this limitation, we extracted all sulcal pits at each stage of the FEM models (from *t*_0_ to *t*_7_), matched them with the corresponding sulcal pits in early‐ and later‐stage MRIs, and aligned them to their respective gestational weeks. Figure 11a illustrates the relationship between gestational weeks and the evolution of the number of sulcal pits. As gestational weeks progress, the number of sulcal pits increases, reflecting the brain's growing complexity in folding and fissure development. This figure demonstrates that FEM models serve as a valuable tool for bridging the gap between two longitudinal datasets. Notably, the figure reveals two distinct stages in the evolution of sulcal pits. During the initial stage, the number of sulcal pits in the FEM models increases in a relatively linear manner. In the second stage, however, the number of sulcal pits stabilizes, with minimal change observed. This transition highlights a key point in cortical development when folding becomes more established, aligning with observed patterns in neuroimaging studies (Im and Grant 2019). This two‐stage development of sulcal pits is evident in the MRI data shown in Figure 11a. In the early gestational weeks, the number of sulcal pits increases rapidly, whereas in the later stages, their numbers stabilize with minimal change. This clear alignment between the FEM models and MRI data underscores the effectiveness of the FEM models in predicting brain folding and the spatiotemporal evolution of sulcal pits. Table S1 presents the number of sulcal pits for all 24 cases at each growth stage (*t*_1_ to *t*_7_), as well as the number of sulcal pits in the corresponding MRI scans at the early and later stages. Figure S6 represents the distribution of sulcal pits across the early and later stages of eight FEM models.

To investigate how sulcal pits establish in the brain model, we extracted the SDSP between all the simulated developmental stages (*t*_1_, *t*_2_, …, *t*_7_) and the early‐stage MRI brain (*t*_0_) of each fetal brain. As the fetal brain develops from *t*_1_ to *t*_7_, we observed a decrease in brain similarity of sulcal pits with *t*_0_ (Figure 11b). The SDSP of two brain models between each developmental stage (*t*_1_, *t*_2_, …, *t*_7_) and *t*_0_ is significantly lower (*p* < 0.001) than the SDSP of two brain models between its previous stage (*t*_0_, *t*_1_, …, *t*_6_) and *t*_0_. However, there is no significant difference between SDSP of *t*_7_ and *t*_0_ and SDSP of *t*_6_ and *t*_0_ (*p* = 0.21). We also observed that the SDSP of the brain models between *t*_7_ and *t*_0_ was significantly higher than that between the later‐stage MRI brain model and the early‐stage MRI scan (at *t*_0_). This discrepancy may be attributed to segmentation issues arising from the compromised quality of the fetal brain MRI scans at the later stages, possibly due to fetal movement. On the other hand, this highlights the advantage of FEM models in reliably predicting the evolution of sulcal pits in the brain. An interesting observation in Figure 11b is that once the folds and fissures are established in the model (between *t*_6_ and *t*_7_), there is minimal change in both the number and locations of the sulcal pits. This indicates their stability during brain growth and folding, a pattern consistent with imaging observations and supporting the notion that sulcal pits can serve as landmarks bridging the prenatal and postnatal stages.

## 4 Discussion

In this study, we developed image‐based true‐scale mechanical models to elucidate how brain folds form during development and how sulcal pits evolve and establish within the folded structure. Our mechanical model demonstrates that the faster‐growing cortical layer overlying the more slowly growing white matter core induces compressive stresses in the cortex, leading to invagination of the cortex into the white matter and the formation of folds (Tallinen et al. 2016). As growth progresses, these folds become increasingly complex, giving rise to secondary and tertiary folds that resemble those observed in the mature brain. The simulations reveal that initial surface undulations (primary folds) during early developmental stages serve as critical locations guiding the formation of later‐developing gyri and sulci. This finding aligns with other modeling studies that, even with simplified geometries, suggest fold positioning is strongly influenced by initial surface undulations (Chavoshnejad et al. 2023). This effect is especially pronounced in the formation and establishment of sulcal pits, as these are the deepest sulcal roots that remain preserved in position despite substantial cortical growth and brain size increase (Meng et al. 2014).

The results of this study indicate that our models are powerful tools for dynamically capturing brain growth and folding processes, as well as for explaining the evolution and conservation of sulcal pits. A primary advantage of these mechanical models lies in their ability to trace the progression of surface morphology and sulcal pits across an arbitrary number of time points, a capacity limited in imaging studies due to the scarcity of longitudinal scans from individual brains (Im and Grant 2019). Thus, these simulations serve as a valuable complement to imaging data, enhancing our understanding of the brain's dynamic developmental trajectory. The results in Figure 8 show that models derived from high‐quality imaging data can capture brain growth, folding, and the evolving patterning of sulcal pits. With reliable early‐stage fetal brain inputs, the model can effectively predict subsequent surface morphology and sulcal pit patterns following growth and folding. This predictive capability represents a significant advancement in forecasting patient‐specific sulcal pit development, especially since sulcal pit patterns are highly sensitive markers for detecting subtle abnormalities that traditional cortical measures, such as gyrification index and curvature, may overlook (Im and Grant 2019).

The simulations indicate that sulcal pits act as anchor points in the folding morphology of the brain. During the brain's expansion and folding, the number of sulcal pits increases, while previously formed pits remain stable, serving as conserved landmarks. Notably, as shown in Figure 11, when the brain is highly folded and coincides with tertiary folding, the number of sulcal pits exhibits only a slight increase, reflecting the establishment of these features. Importantly, these pits are conserved even after birth and throughout infancy (Meng et al. 2014). Consequently, sulcal pits can serve as a valuable proxy for linking prenatal and postnatal periods, while simultaneously addressing the heterogeneity of folding morphologies across individual brains. The models presented in this study can be further extended to replicate postnatal periods, supporting imaging studies that have demonstrated the position and spatial variance of sulcal pits in the fetal brain are similar to those in the adult brain (Yun et al. 2020). In line with these observations, our mechanical models corroborate imaging findings regarding the establishment of sulcal pits in the fetal brain, particularly during the rapid growth of the third trimester, which underscores the stable locations of the earliest cortical sulci. Furthermore, the model's predictions regarding the increasing number of sulcal pits align with in vivo fetal MRI observations, indicating that sulcal emergence occurs during specific time frames in fetal brain development. Together, these findings enhance our understanding of how sulcal pits formation influences brain morphology across developmental stages (Chi et al. 1977; Garel et al. 2001; Fogliarini et al. 2005; Habas et al. 2012).

One interesting observation from Figure 9 is that, in certain instances where the early‐stage and later‐stage MRI scans cannot be matched to their own cases based on SDSP, the FEM model demonstrates its capability in simulating the folding process and preserving sulcal pits. This is evidenced by the higher similarity between the later‐stage FEM model and the corresponding MRI scan from the same fetuses. Such findings suggest that the later‐stage brain model generated using FEM exhibits a more comparable distribution of sulcal pits with the later‐stage MRI scan from the same fetus. The later‐stage FEM model can mimic the progression of sulcal pits throughout growth and folding, although these results are still confounded by geometric imperfections present in fetal MRI scans. This highlights the potential of computational modeling to accurately capture sulcal pit distributions, even in cases where imaging data face quality challenges.

Recently, the accurate timing of sulcal emergence has been utilized as a reliable index for estimating gestational age and detecting developmental disorders (Yun et al. 2022). Abnormal patterns of sulcal pits have been employed to study several brain disorders, including Down Syndrome (Yun et al. 2021), polymicrogyria (Im et al. 2013), “isolated” agenesis of corpus callosum (Tarui et al. 2018), congenital heart disease (Ortinau et al. 2019), and ASD (Brun et al. 2016). Consequently, predictive models show great promise in simulating the development of the brain in relation to these specific disorders. In fact, these models offer the opportunity to manipulate various parameters, thereby elucidating their effects on both normal and disordered brain development. Such possibilities are not feasible with imaging data, which do not allow for the manipulation of growth and geometric parameters to study their contributions to brain development. Therefore, computational models serve as powerful tools for understanding the cause‐and‐effect relationships underlying brain development and disorders, ultimately enhancing our ability to identify and address neurodevelopmental challenges.

In our future studies, we aim to gain a deeper understanding of the mechanisms underlying brain disorders characterized by atypical development of the spatiotemporal evolution of sulcal pits. Our long‐term hypothesis posits that abnormal patterns of sulcal pits observed after birth originate from atypical development during the fetal stage. One potential candidate for this investigation is ASD, which manifests atypical sulcal pit patterning during infancy and later stages (Brun et al. 2016; Auzias et al. 2014). Given that ASD symptoms typically emerge after birth, primarily during infancy and childhood, there is limited knowledge regarding its development during the fetal stage. Nevertheless, based on the atypical establishment of sulcal pits observed postnatally in ASD, we speculate that the disorder may also develop abnormally during the fetal period. Consequently, modeling sulcal pits could serve as a proxy to link prenatal and postnatal stages of development. Investigating the development of sulcal pits in ASD, a heterogeneous disorder primarily attributed to genetic factors, may help clarify the numerous contradictory findings related to alterations in cortical layer thickness, surface area, volume, and gyrification index associated with ASD (Mensen et al. 2017; Ohta et al. 2016; Khundrakpam et al. 2017; Smith et al. 2016; Hardan et al. 2006). Therefore, we propose that examining the mechanics of typical sulcal pits development provides a valuable foundation for further studies on the development of ASD.

Finally, akin to other modeling studies, our research acknowledges certain simplifications and limitations inherent in computational models. In this study, we focused exclusively on differential tangential growth, which is a complex phenomenon that may arise from various underlying processes. However, other significant contributing factors, such as the impact of neural connectivity and its role in cortical development, were not incorporated into our model (Essen 1997; Chavoshnejad et al. 2021; Holland et al. 2015; Wang et al. 2024; Solhtalab et al. 2025). The material properties utilized in our simulations were assumed to be constant. Yet, existing literature indicates that the mechanical properties of both the cortical plate and white matter can evolve throughout development, affecting the overall mechanical behavior of the brain (Walter et al. 2023). This variability underscores the importance of integrating time‐dependent material properties in future modeling efforts to enhance accuracy. Furthermore, our current models did not account for hemispheric asymmetries or sex differences, both of which have been shown to influence the morphology of sulcal pits in fetal brains (Yun et al. 2022). These factors could contribute to variability in the development of brain structures, indicating that future studies should explicitly incorporate these parameters to facilitate a more nuanced discussion of their effects. In this study, sulcal pits were extracted using the concept of sulcal depth; however, previous research has also employed mean curvature as an extraction method. While both approaches are applicable to fetal brain analysis and yield comparable results, discrepancies persist between the two methods (Yun et al. 2020). Establishing a reliable geometric feature for sulcal pits extraction could provide a clearer understanding of these differences and improve the robustness of our findings.

In summary, the limitations and simplifications discussed in this study open avenues for future work to better align models with imaging data. These efforts include developing more accurate models, acquiring high‐resolution fetal MRI scans, and improving sulcal pit extraction algorithms. Despite the current challenges, the proposed framework holds promise as a hypothesis‐testing tool to investigate the effects of mechanical factors, such as tissue stiffness, on brain growth and folding. Ultimately, addressing these limitations will strengthen future modeling studies and enhance our ability to explore the complexities of brain development.

## 5 Conclusion

In this study, we developed and evaluated the first image‐based true‐scale mechanical model to investigate the spatiotemporal evolution of brain sulcal pits in individual fetal brains. Constructed using scans from the initial time point of longitudinal data, our model predicts the brain's surface morphology by comparing sulcal pits between model predictions and later time point scans. This dynamic approach simulates the transformation of a smooth fetal brain with primary folds into a convoluted morphology. Our findings align with imaging data, demonstrating that sulcal pits remain stable during brain development and can serve as crucial markers linking prenatal and postnatal brain characteristics. The main advantage of the mechanical models lies in their ability to trace the progression of surface morphology and sulcal pits across an arbitrary number of time points, a capacity limited in imaging studies due to the scarcity of longitudinal scans from individual brains. The developed model provides a platform for studying the evolution of sulcal pits in both healthy and disordered brains, which is particularly significant given that altered patterns of sulcal pits are observed in disorders. Ultimately, our study lays a foundation for future investigations into the complex mechanisms driving brain morphology and their implications for understanding neurodevelopmental challenges.

## Author Contributions

**A.S.:** methodology, formal analysis, software, writing – original draft. **Y.G.:** methodology, formal analysis, software, writing – original draft. **A.G.:** formal analysis, writing – review and editing. **W.D.:** investigation, supervision, writing – review and editing, funding acquisition. **M.J.R.:** conceptualization, investigation, supervision, writing – review and editing, funding acquisition. All authors reviewed the manuscript.

## Conflicts of Interest

The authors declare no conflicts of interest.

## Supporting information


**Data S1:** Supporting Information.

**Video S1:** A mechanical model of human brain growth and folding. The video illustrates how a smooth fetal brain gradually expands and develops folds step by step.

## Data Availability

The data that support the findings of this study are available from the corresponding author upon reasonable request.
