# Supplementary material for: Mechanics of the Spatiotemporal Evolution of Sulcal Pits in the Folding Brain
Source: Hum Brain Mapp. 2025 Aug 27;46(13):e70332. doi: 10.1002/hbm.70332 (PMC12381649; doi:10.1002/hbm.70332)
Supplement: Supplementary file 1 — Data S1: Supporting Information. [file HBM-46-e70332-s001.docx]

**Supplementary Information**

**Mechanics of the Spatiotemporal Evolution of Sulcal Pits in the Folding Brain**

Akbar Solhtalab^1+^, Yanchen Guo^2+^, Ali Gholipour^3,4,5^, Weiying Dai^2^, Mir Jalil Razavi^1*^

^1^Department of Mechanical Engineering, State University of New York at Binghamton, Binghamton, NY, USA

^2^Department of Computer Science, State University of New York at Binghamton, Binghamton, NY, USA

^3^Department of Radiological Sciences, University of California Irvine, Irvine, CA, USA

^4^Department of Electrical Engineering and Computer Science, University of California Irvine, Irvine, CA, USA

^5^Department of Radiology, Boston Children’s Hospital, Boston, MA, USA

^+^These authors contributed equally to this work.

*Corresponding Author: [mrazavi@binghamton.edu](mailto:mrazavi@binghamton.edu)


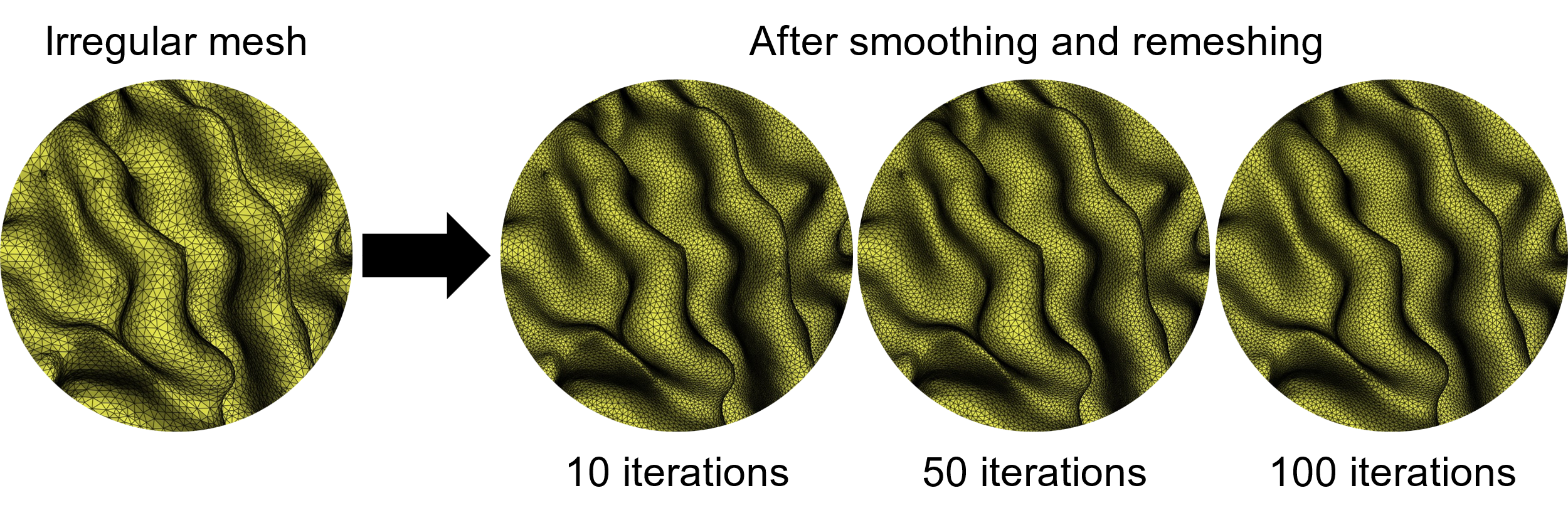


**Fig. S1.** Effect of Taubin smoothing iterations on white matter surface quality. White matter surfaces were smoothed using 10, 50, and 100 iterations of Taubin smoothing to assess the impact on surface geometry. Visual inspection shows that 100 iterations produce a smooth and anatomically consistent surface while preserving key sulcal and gyral features. This setting was selected for all models to ensure optimal mesh quality for FEM simulations.


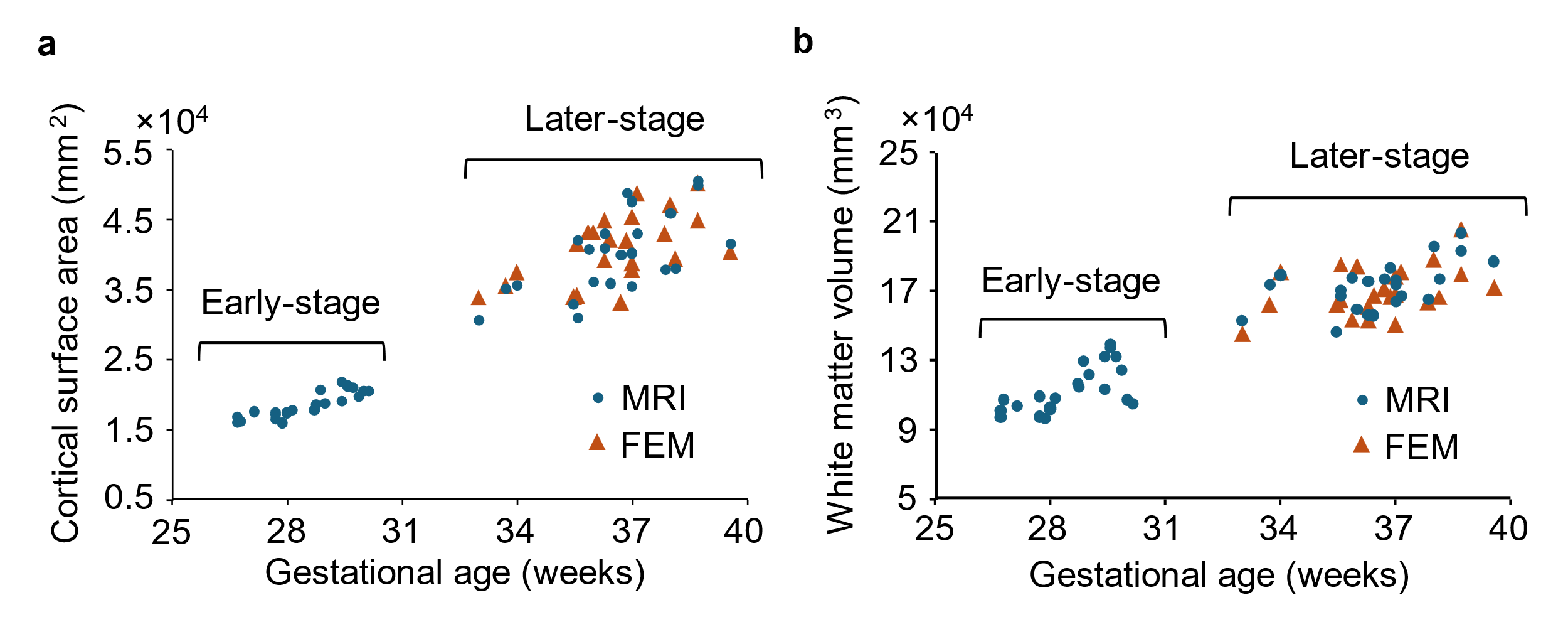


**Fig. S2.** Cortical surface area and white matter volume growth across gestational age from MRI and FEM simulations. Each point represents a fetal subject with MRI measurements taken at two developmental stages. **a** Cortical surface area and **b** white matter volume are plotted against gestational age. Triangle points represent FEM simulation results, which closely match MRI data at the later time point, validating the chosen growth parameters.

**
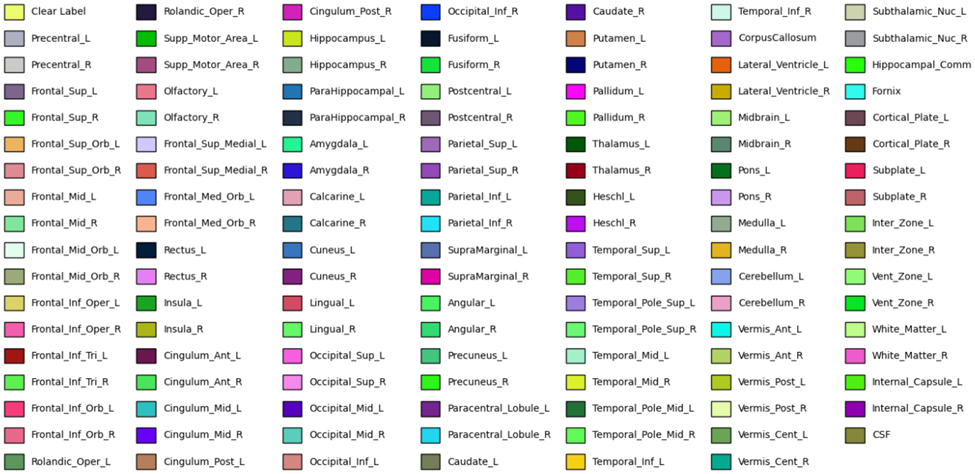
**

**Fig. S3.** Color map of cortical parcellations. Each color represents to a distinct cortical region and corresponds to the background colors used in Figs. 3 through 11.


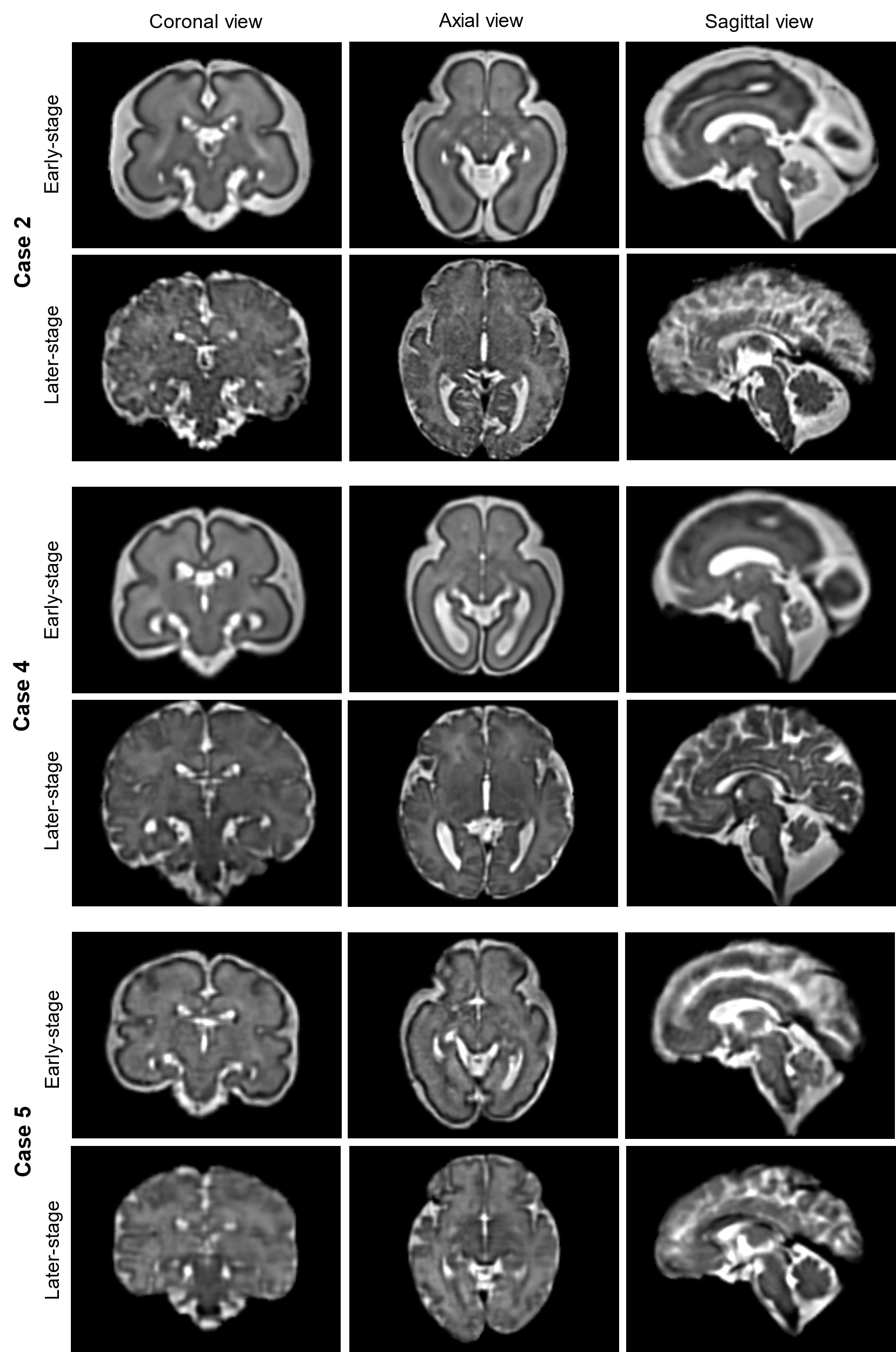


**Fig. S4.** Orthogonal views of T2-weighted MR images in atlas space for representative cases shown in Fig. 9 of the main text. Coronal, axial, and sagittal slices are presented to illustrate the anatomical quality of the reconstructed images. These views complement the main figures by providing additional spatial context for the surface reconstructions and sulcal pit analysis.


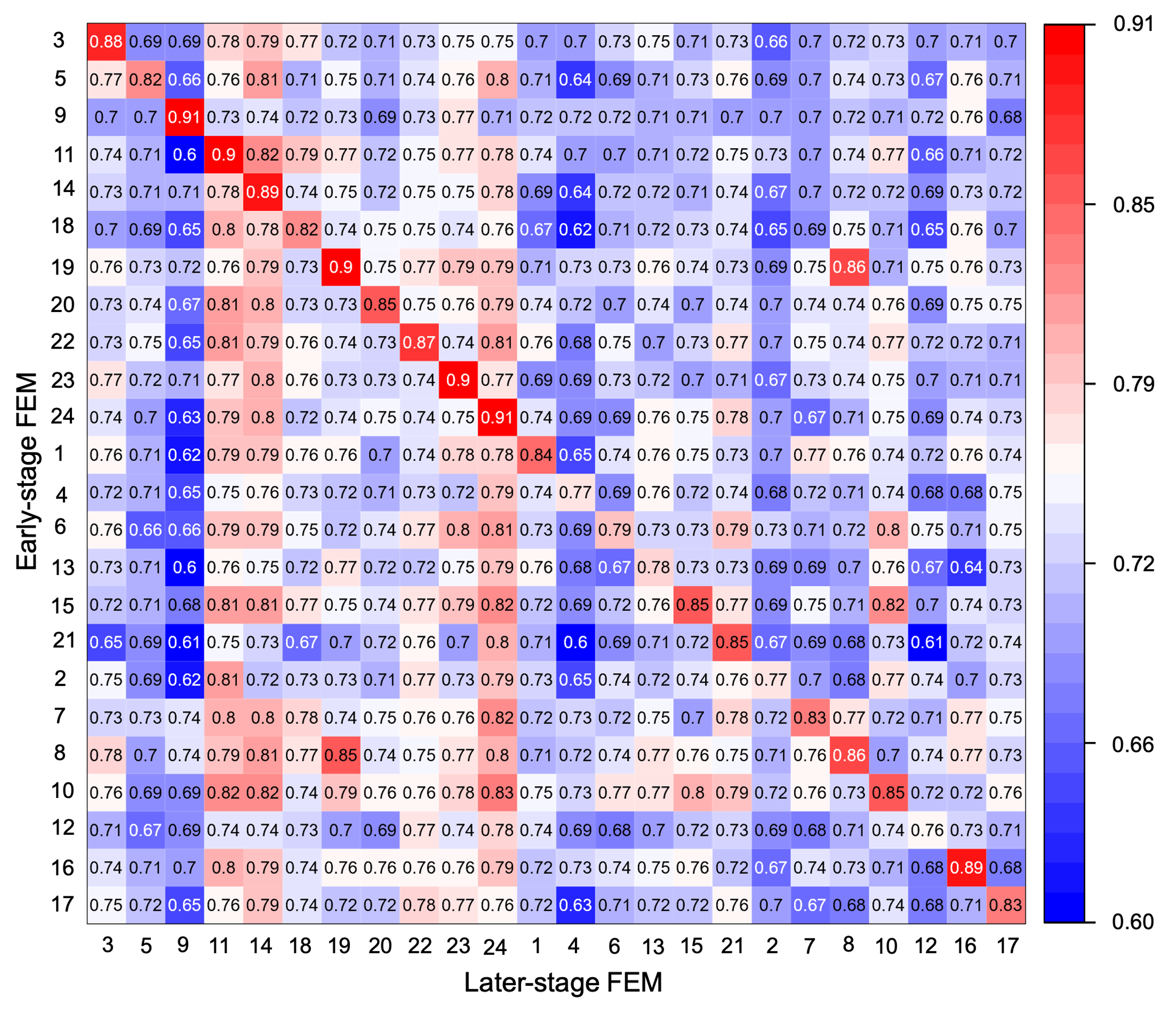


**Fig. S5.** Sulcal pits-based similarity (SDSP) between early-stage FEM and later-stage FEM for all subject pairs. The diagonal indicates similarity within the same subject, while the off diagonal reflects similarity between different subjects.

**Table S1.** Number of sulcal pits for all 24 cases at each growth stage (t_1_ to t_7_), as well as the number of sulcal pits in the corresponding MRI scans at the early and later stages.

| **Case**  **number** | **t_0_**  **MRI early-stage**  (same for FEM) | **t_1_** | **t_2_** | **t_3_** | **t_4_** | **t_5_** | **t_6_** | **t_7_** | **MRI Later-stage** |
| --- | --- | --- | --- | --- | --- | --- | --- | --- | --- |
| 1 | 91 | 93 | 97 | 104 | 111 | 113 | 117 | 127 | 118 |
| 2 | 58 | 66 | 78 | 87 | 96 | 97 | 103 | 106 | 134 |
| 3 | 89 | 95 | 105 | 110 | 113 | 117 | 121 | 111 | 127 |
| 4 | 61 | 83 | 108 | 113 | 120 | 123 | 124 | 125 | 136 |
| 5 | 70 | 80 | 92 | 99 | 99 | 96 | 106 | 104 | 109 |
| 6 | 58 | 63 | 78 | 85 | 96 | 105 | 107 | 114 | 123 |
| 7 | 67 | 75 | 93 | 103 | 112 | 104 | 104 | 102 | 113 |
| 8 | 69 | 76 | 88 | 97 | 103 | 103 | 101 | 106 | 109 |
| 9 | 104 | 106 | 107 | 108 | 110 | 112 | 113 | 113 | 121 |
| 10 | 35 | 34 | 42 | 56 | 69 | 75 | 84 | 95 | 106 |
| 11 | 64 | 75 | 103 | 92 | 100 | 101 | 103 | 103 | 104 |
| 12 | 79 | 91 | 108 | 116 | 126 | 122 | 124 | 124 | 124 |
| 13 | 60 | 78 | 93 | 100 | 107 | 116 | 119 | 119 | 138 |
| 14 | 86 | 97 | 99 | 110 | 120 | 120 | 125 | 122 | 124 |
| 15 | 62 | 71 | 80 | 91 | 96 | 103 | 92 | 96 | 112 |
| 16 | 79 | 82 | 94 | 101 | 104 | 111 | 115 | 126 | 106 |
| 17 | 63 | 68 | 80 | 80 | 86 | 97 | 101 | 105 | 114 |
| 18 | 80 | 87 | 95 | 106 | 108 | 108 | 110 | 112 | 121 |
| 19 | 76 | 80 | 84 | 90 | 92 | 96 | 98 | 105 | 109 |
| 20 | 73 | 80 | 89 | 95 | 95 | 99 | 102 | 102 | 106 |
| 21 | 69 | 81 | 89 | 88 | 92 | 94 | 100 | 104 | 128 |
| 22 | 67 | 77 | 94 | 90 | 106 | 110 | 110 | 109 | 102 |
| 23 | 83 | 92 | 100 | 104 | 109 | 114 | 109 | 118 | 122 |
| 24 | 59 | 70 | 80 | 97 | 105 | 108 | 193 | 123 | 106 |

| 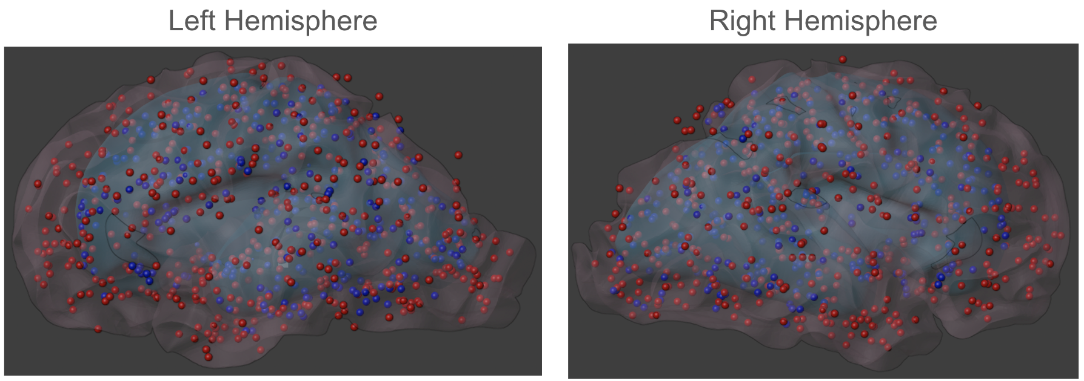 | 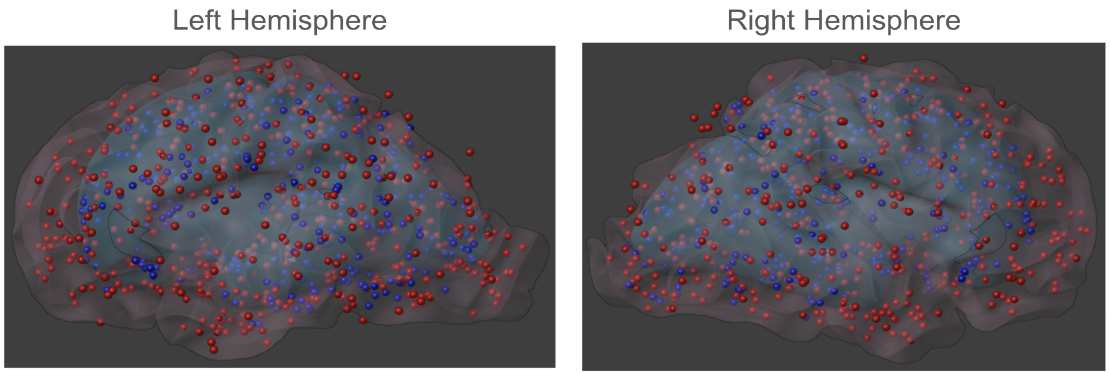 |
| --- | --- |
| Left Hemisphere | Right Hemisphere |

**Fig. S6.** Distribution of sulcal pits across the early and later stages of eight brain growth models. The red surface represents the later-stage FEM, while the blue surface corresponds to the early-stage FEM. Red dots denote the sulcal pits at the later stage, while blue dots indicate the corresponding sulcal pits at the early stage.


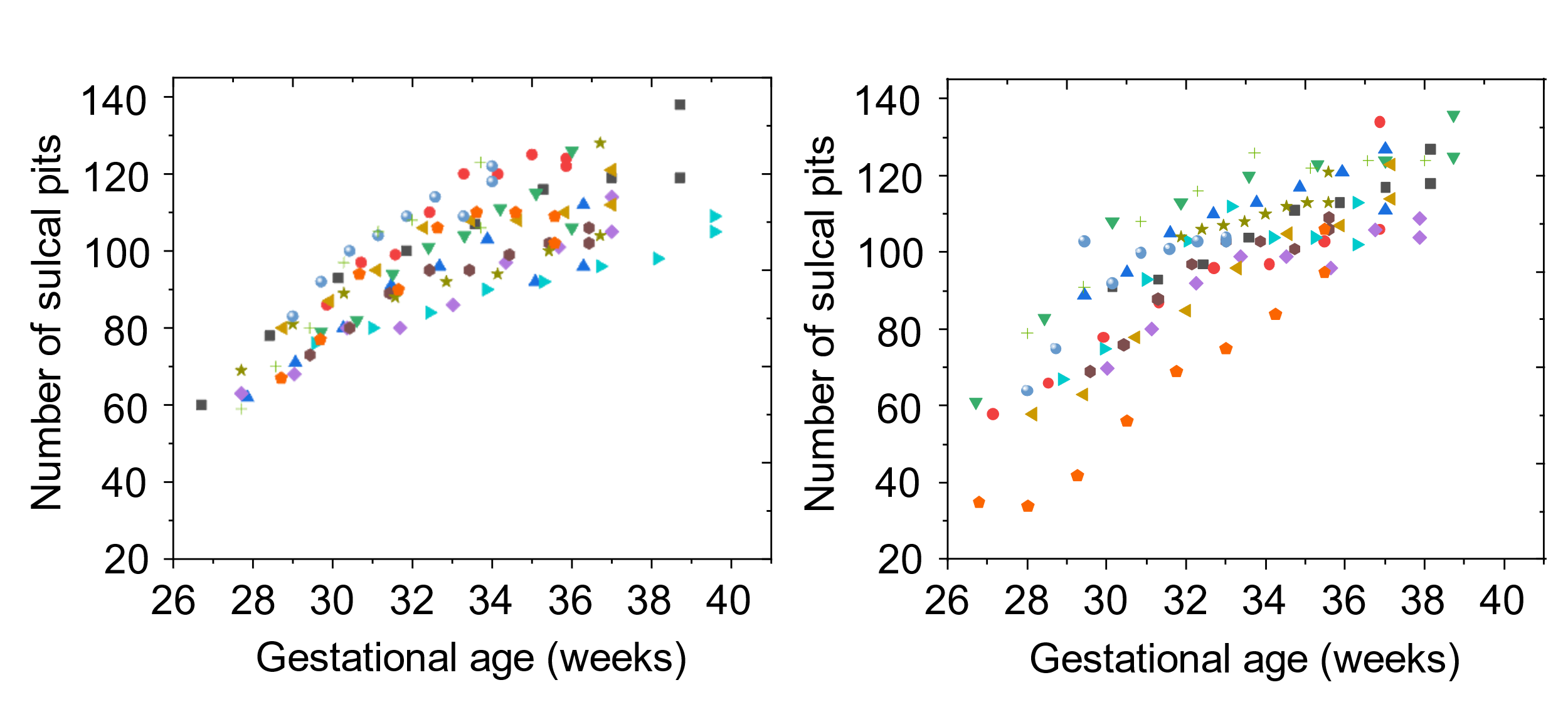


**Fig. S7.** Evolution of sulcal pits across gestational age for individual subjects. Each figure displays 12 subjects, with each subject represented by a distinct symbol and color to clearly track their trajectory.
